# Enablers and barriers to human papillomavirus vaccination implementation among key stakeholders in Ethiopia: A qualitative study

**DOI:** 10.1371/journal.pone.0361925

**Published:** 2026-11-02

**Authors:** Kibir Temesgen Assefa, Abebaw Gebeyehu Worku, Achenef Asmamaw Muche, Bisrat Misganaw Geremew, Mulat Adefris Woldetsadik, Kassahun Alemu

**Affiliations:** 1 Reproductive Health Department, Institute of Public Health, College of Medicine and Health Sciences, University of Gondar, Gondar, Ethiopia; 2 Midwifery Department, College of Medicine and Health Sciences, Wollo University, Dessie, Ethiopia; 3 Department of Epidemiology and Biostatistics, Institute of Public Health, College of Medicine and Health Sciences, University of Gondar, Gondar, Ethiopia; Regional Health Care and Social Agency of Lodi, ITALY

## Abstract

**Introduction:**

Human papillomavirus (HPV) vaccination is an effective and lifesaving intervention against cervical cancer. However, achieving high, equitable, and sustainable uptake remains a significant challenge in low-resource settings, including Ethiopia. Therefore, this study aimed to explore the enablers and barriers to HPV vaccination implementation in Ethiopia.

**Methods:**

An interpretive descriptive qualitative design was employed in purposively selected settings representing diverse socio-ecological contexts (Afar, Amhara, Addis Ababa, and Dire Dawa). Six focus group discussions were conducted with adolescent girls (n = 14), parents of eligible adolescent girls (n = 14), and community representatives (n = 14). In addition, nineteen key informant interviews (KIIs) were conducted with healthcare providers, Expanded Programme on Immunization (EPI) coordinators, and technical advisors from governmental and non-governmental organizations. Data were analysed using a combined deductive–inductive content analysis guided by the Consolidated Framework for Implementation Research (CFIR 2.0).

**Results:**

Multiple interacting factors influenced HPV vaccination implementation in Ethiopia, categorised as enablers and barriers across community, health system, and policy levels. Enablers included the Perceived Vaccine Attributes and Delivery Adaptability, Supportive Policy and Multisectoral Strategic Partnerships, Socio-cultural and Belief-based Drivers, and Health System Readiness and Workforce Motivation. However, several barriers limited implementation, including Suspicions About Targeting 9–14-year-old Girls and Consent-related Administrative Obstacles, Pervasive Misinformation and Cognitive Resistance, Health System Resource Constraints and Coordination Gaps, Donor Dependency Risks and Financial Sustainability Concerns, and Structural Access Constraints and Equity Gaps.

**Conclusions:**

While policy shifts, external strategic partnerships, and frontline worker dedication have accelerated vaccine reach, the long-term sustainability of Ethiopia’s HPV vaccination program remains constrained by multi-level barriers. Progress toward the global 2030 cervical cancer elimination targets requires trust-building risk-communication strategies to counter fertility myths, simplified consent procedures, targeted domestic resource mobilization, and context-specific delivery models tailored to underserved pastoralist and urban sub-populations.

## Introduction

Human papillomavirus (HPV) vaccination is recognised as the most efficient and effective public health intervention against cervical cancer and other HPV-related diseases [1]. The implementation of HPV vaccination, through routine immunisations or school-based campaigns, has been steadily increasing, with 147 countries having introduced it as of 2024 [2]. Effective implementation of HPV vaccination in high-income countries (HICs) has led to significant reductions in cervical cancer incidence and deaths. However, low- and middle-income countries (LMICs), which account for 94% of cervical cancer-related deaths, are struggling to implement it effectively [1,3]. Despite its introduction in most countries, effective, equitable, and sustainable implementation remains a challenge, particularly in LMICs. In 2020, only one-fourth of LMICs included HPV vaccination in their national immunisation programmes, compared to more than 85% of HICs [1,4,5].

In Ethiopia, HPV-related cervical cancer is one of the leading causes of cancer-related morbidity and mortality among women [6]. Its burden increased substantially between 2010 and 2020, with annual cases rising from 4,648 to 7,445 and deaths from 3,235 to 5,338, while the number of women aged ≥15 years at risk increased from 20.9 to 36.9 million [7–9]. The estimated annual incidence and mortality rates were 35.9 and 22.6 per 100,000 women, respectively, among the highest in the region [10]. In response, the country introduced HPV vaccination in 2018 with a two-dose schedule targeting 14-year-old girls. In 2024, it transitioned to a single-dose schedule for girls aged 9–14 years through a nationwide multi-age cohort campaign [11]. Through this campaign, Ethiopia has successfully delivered the vaccine to over 6.8 million girls, increasing the total number of vaccinated girls to at least 13 million in 2024 [12]. Although this policy decision is an important step towards cancer prevention, the country is still struggling to achieve the goal of vaccinating 90% of girls by 2030 [13–15].

The implementation of HPV vaccination in Ethiopia occurs within complex social, cultural, and institutional environments [8,16]. It is mainly delivered through school-based programmes, supplemented by outreach for out-of-school girls, with implementation varying across settings due to differences in access, awareness, and sociocultural factors [17]. Reported HPV vaccination coverage has varied across geographic and social contexts [18]. Challenges in reaching all eligible girls, particularly those out of school, indicate that implementation processes may affect program performance and equity [19]. Vaccinating adolescents involve multiple actors, including health workers, educators, parents, community leaders, and the girls themselves [20]. Program delivery is also influenced by perceptions of vaccine safety and purpose, community norms related to age and sexuality, trust in health and government institutions, and the capacity of health and education systems to coordinate effectively [6,13,15,17]. These factors highlight the need for qualitative inquiry to understand how stakeholders interpret, experience, and respond to HPV vaccination initiatives within their local contexts.

A growing body of evidence suggests that successful implementation of HPV vaccination in Ethiopia could substantially reduce the future burden of cervical cancer and avert significant health and economic losses [8]. Modelling studies estimate that routine HPV vaccination could avert up to approximately 970,000 cervical cancer cases and save nearly 932,000 lives over the coming decades in Ethiopia compared to a no-vaccination scenario, while also reducing catastrophic out-of-pocket medical expenses for households [21,22]. These figures highlight that successful HPV vaccine implementation is critical not only for reducing morbidity and mortality from cervical cancer but also for promoting health equity and financial protection in Ethiopia’s resource-limited settings.

Despite the substantial public health benefits of HPV vaccination, critical gaps remain in understanding the contextual and implementation-related barriers affecting its effective delivery in Ethiopia. There is a scarcity of studies examining the interconnected socio-cultural, health system, operational, and contextual factors influencing HPV vaccination implementation. Existing studies have given little attention to influential stakeholders in the implementation process such as health administrators and community representatives (religious and community leaders) [6,13–15,17]. Additionally, previous quantitative analyses conducted as part of this project found that urban adolescent girls were more likely to remain unvaccinated against HPV than their rural counterparts [23]. However, the underlying reasons for this counterintuitive finding are not sufficiently explained. These disparities indicate the influence of complex contextual factors that quantitative methods alone do not capture.

Therefore, this study aimed to explore the enablers and barriers to HPV vaccination implementation in Ethiopia.

## Methods

### Study setting and period

The study was conducted in purposively selected regions and city administrations of Ethiopia, including Afar, Amhara, Addis Ababa, and Dire Dawa. These sites were purposively selected based on prior quantitative findings from the parent study, which flagged high concentrations of unvaccinated adolescents. The selected settings also represent diverse socio-ecological, infrastructural, and livelihood contexts that directly influence vaccine delivery. Afar region represents a pastoralist setting, characterised by nomadic populations, dispersed settlements, limited static health infrastructure, and severe logistical challenges for routine outreach delivery. Amhara region reflects a largely rural, agrarian health delivery context, which includes conflict-affected clusters where routine primary healthcare services have experienced intermittent operational disruptions. Addis Ababa and Dire Dawa represent densely populated urban environments with established healthcare infrastructure, high school-enrolment, and widespread access to mass media, and private educational facilities [24]. Data were collected between 10 September and 30 November 2025 from health facilities, non-governmental organisations, and communities involved in HPV vaccine delivery.

### Study design

We employed an interpretive descriptive (ID) design because it is specifically intended for applied health contexts, where findings should be transferable to clinical or public health practice. It enables researchers to answer not only what, but also how and why questions, interpret social, cultural, and health system complexities, identify underlying patterns, and generate context-specific recommendations. Epistemologically, this study is grounded in constructivist and relativist ontologies, which recognize that implementation experiences are subjective, pluralistic, and co-constructed through continuous interactions between stakeholders and their institutional environments [25]. This study was reported in accordance with the Consolidated Criteria for Reporting Qualitative Research (COREQ) guidelines [26]. The completed 32-item COREQ checklist is provided as Supporting Information (S3 File).

**Relationship to companion studies.** This qualitative study forms part of a broader doctoral research project on HPV vaccination among adolescent girls in Ethiopia. It was informed by findings from our companion quantitative studies [18,23], particularly the observed higher non-uptake of HPV vaccine among adolescent girls in urban settings. These findings provided the rationale for further exploring contextual and implementation-related factors contributing to urban non-uptake. While the companion studies examined quantitative aspects of HPV vaccine non-uptake and its determinants, the present study explores stakeholders’ perspectives on implementation-related enablers and barriers. Although all studies are part of the same broader research project, they address distinct research questions and use different analytical approaches and outcomes. There is no overlap in the qualitative data, participant quotations, analyses, or findings reported in the present manuscript.

### Participants selection, sampling technique and sample size

For the focus group discussions (FGDs), heterogeneous purposive sampling was used to maximise variation in participants’ socio-demographic characteristics, roles, and experiences. Participants included adolescent girls, parents of eligible adolescent girls, and community and religious leaders. FGD composition was purposively determined according to the stakeholder group and study objectives. FGDs 1 and 2 comprised adolescent girls, FGDs 3 and 4 comprised male and female parents, and FGDs 5 and 6 comprised religious and community leaders. This composition enabled the study to capture diverse perspectives across the different stakeholder groups.

Adolescent girls were identified through schools with the support of school directors. Girls aged 17–18 years were purposively selected, based on their age and grade, to explore perspectives informed by their prior eligibility for, or potential exposure to, the earlier HPV vaccination programme. These adolescents were expected to have prior experience of HPV vaccination, having been eligible at age 14 under Ethiopia’s initial single-age vaccination strategy, and to articulate their perspectives on vaccination barriers and facilitators more clearly than younger adolescents. Within the selected schools, participants were further purposively recruited based on their willingness to take part, good academic performance, and involvement in school clubs and mini-media activities. This purposive approach was intended to identify information-rich participants rather than achieve statistical representativeness.

For the key informant interviews (KIIs), participants were selected using expert purposive sampling based on their experience and expertise in HPV vaccination implementation. These included immunization experts and technical advisors at the Ministry of Health, staff from development partner organisations, regional, woreda and zonal immunisation coordinators, hospital administrators, and healthcare providers responsible for immunisation services. Key informants were identified in consultation with the relevant health authorities to ensure the inclusion of individuals with appropriate roles and experience in HPV vaccine rollout.

The final sample comprised 42 discussants distributed across six distinct FGDs, and 19 key informants selected for KIIs. All planned samples were successfully recruited. Although three key informants had competing personal schedules that initially conflicted with the planned interview times, sessions were rescheduled to accommodate their availability. Two planned KII participants were replaced after being unavailable on two consecutive occasions despite rescheduling attempts.

### Data collection tools

The FGD and KII guides were developed based on the study objective, a review of relevant literature, and existing similar tools [6,13–15,17]. The guides were further informed by updated domains and constructs of CFIR [27]. Constructs relevant to the study setting and local context were selected to ensure contextual appropriateness. Both guides were initially prepared in English, translated into Amharic, and then back-translated into English by language and subject matter experts to ensure consistency. Experts in public health and qualitative research reviewed the guides to assess their content. Minor revisions were made to improve wording, sequencing, and contextual relevance. The guides were piloted and refined for clarity, relevance, and cultural appropriateness.

### Data collection procedures

FGD participants were grouped by their socio-demographic characteristics and roles in the community. The principal investigator (PI) and trained assistant moderators facilitated the discussions. Each participant was assigned a number, sat in a circle, and answered questions in a round-robin format, where participants spoke in turn to ensure equal opportunities to speak and to minimise dominance or passivity. The moderators used probing questions to encourage greater engagement and prompt participants to elaborate on their ideas. Note takers took notes and audio-recorded the discussions using smartphones after obtaining consent. FGDs were conducted face to face in school classrooms for students, health center meeting halls for parents and in kebele (the smallest administrative unit in Ethiopia) administration halls for community representatives (community and religious leaders), after communicating with the respective authorities. During the discussions, participants were allowed to speak freely, and respecting others’ ideas was an established FGD norm. Each session lasted 60–120 minutes.

The PI conducted KIIs both in person (face to face) and virtually, in clinics for providers and offices for administrators or coordinators, after working hours. Each interview lasted 30–69 minutes and was audio-recorded. Probing questions were used to elicit additional information.

All Interviews were conducted in Amharic as it is the official language in Amhara, Addis Ababa and Dire Dawa, and all participants were able to speak it. In the Afar Region, participants who were able to speak in both Afar and Amharic were purposively selected to facilitate communication while preserving the contextual meaning of their experiences. Conducting the FGDs in Amharic among bilingual participants minimized the need for multiple translation stages (i.e., Afar to Amharic and subsequently to English), thereby reducing the potential loss or distortion of culturally embedded expressions, nuances, and intended meanings. It also enabled the moderator to interact directly with participants, probe responses, and clarify meanings during the discussions.

Audio recordings were transcribed verbatim in Amharic and subsequently translated into English. Translation accuracy was enhanced through verification against the original audio recordings, review of ambiguous passages, and comparison with field notes. Coding was conducted using the English transcripts, while participant quotations were cross-checked against the original recordings and Amharic transcripts to ensure that their intended meanings and contextual nuances were preserved. This approach helped reduce the translation burden while enhancing the credibility, consistency, and contextual integrity of the qualitative data.

Saturation was assessed iteratively by the research team throughout data collection and analysis. After each interview or focus group discussion, the team reviewed emerging codes, categories, and meanings and assessed whether new data were generating substantially new information. Both code saturation (no substantially new codes) and meaning saturation (no substantial new insights or dimensions within existing codes) were considered. Saturation was assessed separately for FGDs and KIIs. For FGDs, saturation was considered reached when successive discussions generated no substantially new codes, themes, or perspectives. For KIIs, saturation was considered reached when consecutive interviews yielded no new relevant concepts or insights. Saturation was monitored through ongoing coding, analytic notes, and regular review of emerging findings during the data collection process.

### Reflexivity and positionality

All authors are males. The PI, who conducted the KIIs and led the analysis, is an Assistant Professor and PhD candidate in Reproductive Health at the University of Gondar. AGW is a Senior Health Systems Strategic Advisor at John Snow, Incorporated (JSI), while AAM, BMG, MAW, and KA are Senior researchers at the University of Gondar, College of Medicine and Health Sciences, Institute of Public Health. The authors have advanced academic and professional training in Public Health, with extensive experience in qualitative research. Participants were introduced to the PI as a student conducting a study as part of doctoral research and need the data for research purpose only. Before data collection, the PI recognized that his professional experience and prior knowledge of HPV vaccination could influence interactions with participants and interpretation of their accounts. So, the PI maintained reflexive notes and analytic memos throughout data collection and analysis to critically examine personal assumptions and their potential influence on data interpretation. Discussions with the research team were also used to challenge emerging interpretations and consider alternative explanations. These practices supported a reflexive approach to data collection and analysis and helped minimize the influence of researcher assumptions on the findings.

### Data analysis procedure

Audio transcripts were transcribed in Amharic and translated into English. Transcriptions were verified against audio files and field notes for accuracy, with unclear sections revisited and corrected. Transcripts were imported into Open Code software to facilitate data management, including coding, category development, and storage, while the analysis was conducted solely by the researchers. Data were analysed using a combined deductive–inductive content analysis guided by CFIR 2.0. The analysis was conducted at the level of meaning units, defined as segments of text containing a distinct idea or concept relevant to the study objectives. First, a preliminary deductive coding framework was developed from the relevant CFIR 2.0 domains and constructs, with the operational definitions of the constructs used to guide the interpretation and classification of participants’ accounts. Transcripts were then read repeatedly to gain an overall understanding of the data, after which relevant meaning units were identified, condensed, and assigned to the most appropriate CFIR 2.0 construct. When a meaning unit addressed more than one implementation issue, the researchers considered its context and primary meaning when determining the most appropriate construct. Texts that were not adequately captured by predefined CFIR 2.0 constructs were inductively coded and subsequently refined and grouped into related subcategories and categories.

The coding framework was piloted on a subset of transcripts and iteratively refined throughout the analysis as new concepts emerged or distinctions between codes became clearer. The researchers continuously compared coded segments within and across transcripts to identify similarities, differences, relationships, and patterns within each CFIR 2.0 domain and across stakeholder groups. Two researchers independently coded two full transcripts and compared their coding; discrepancies were discussed in relation to the transcript context and CFIR 2.0 construct definitions and resolved through consensus. The remaining transcripts were then coded by two coders using the refined framework, with ongoing discussion among the research team when ambiguity or new concepts arose. Finally, the coded data were systematically reviewed and synthesized to develop subcategories and main categories, while retaining both CFIR 2.0-informed and inductively derived concepts. This process was intended to enhance analytic consistency, transparency, and reflexivity rather than to calculate a quantitatively defined intercoder reliability coefficient (Table 1).

**Table 1:** Example of the combined deductive–inductive content analysis process, showing meaning units, condensed meaning units, codes, subcategories, and main categories among key stakeholders in Ethiopia, 2025.

Meaning unit (Verbatim quote)	Condensed meaning unit	Code	Subcategory	Main category
“HPV vaccination is recognized as the most effective intervention for preventing cervical cancer and save women’s lives.” (KII-1)	HPV vaccine recognised as most effective for cervical cancer prevention.	Perceived high effectiveness	Evidence-based effectiveness	Perceived Vaccine Attributes and Delivery Adaptability
“School-based delivery helps easy identification of girls using class rosters, and mothers do not need to leave work to go to health facilities.” (KII-1)	School delivery simplifies identification of girls and avoids work disruption.	Easy identification of eligible girls	Relative advantage	
“The HPV vaccine is easier to administer and more culturally acceptable than screening, which is invasive and exposes private parts.” (FGD2P5)	Vaccine preferred over invasive and privacy-exposing screening.	-Easy to administer -Less invasive	Relative advantage	

### Rigor and trustworthiness

To ensure qualitative trustworthiness, strategies were applied across four domains:

**Credibility:** Credibility was enhanced through prolonged engagement with the study context and participants. Before data collection, the PI completed a one-month internship attachment at the Ministry of Health-Ethiopia, which provided an opportunity to become familiar with the study context and establish rapport and interactions with relevant Ministry of Health stakeholders who later participated in key-informant interviews. This was complemented by repeated interactions with participants during data collection, which facilitated rapport building and a deeper understanding of their experiences. Credibility was further strengthened through data-source triangulation across diverse stakeholder groups and member checking. Preliminary conceptual summaries and emerging interpretations were shared with selected key informants, who provided feedback on their accuracy and relevance. Their feedback was used to clarify meanings, refine codes, and adjust emerging interpretations.

**Dependability:** Dependability was supported through a detailed audit trail comprising raw audio recordings, verbatim Amharic and English transcripts, comprehensive field notes, and systematic documentation of coding and analytical decisions.

**Confirmability:** Confirmability was strengthened through explicit bracketing of researchers’ preconceptions, extensive use of verbatim participant quotations, and independent parallel coding of transcripts by two researchers to reduce individual interpretation bias.

**Transferability:** Transferability was supported through detailed descriptions of the study context, health system, participant characteristics, and perspectives, supplemented by illustrative verbatim quotations to enable assessment of applicability to similar settings.

### Ethical approval

The study received ethical approval from the institutional review board (IRB) of the University of Gondar, College of Medicine and Health Sciences, Institute of Public Health (Reference: CMHSSH-UOG IRERC/43/05/2024). An official letter was also sent to the relevant authorities before data collection. Prior to each interview or focus group discussion, participants were informed about the purpose and procedures of the study, the voluntary nature of participation, confidentiality, potential risks and benefits, and their right to decline participation or withdraw at any time without consequence. Considering the geographical dispersion of the study settings and the virtual mode of most KIIs, the written consent information was read to KII participants, and their verbal informed consent was obtained and audio-recorded before each interview. The approved study protocol included a provision for obtaining audio-recorded verbal consent. For FGDs, informed consent/assent was obtained individually from each participant before the discussion, and participants were given an opportunity to ask questions. For participants aged 17 years (minors), guardian consent and adolescent assent were obtained, whereas participants aged 18 years provided informed consent independently. All audio records and study data were de-identified and password-protected, and interviews and FGDs were conducted in private settings to protect participants’ confidentiality.

## Results

### Sociodemographic characteristics of study participants

A total of 42 discussants participated in the six FGDs, with ages ranging from 17 to 57 years. FGD1 and FGD2 included adolescent girls who were secondary school students. FGD3 and FGD4 included parents employed as teachers, merchants, housewives, and a guard. FGD5 and FGD6 included community representatives (religious and community leaders). Participants were from Amhara, Afar, Addis Ababa, and Dire Dawa regions/city administrations. Regarding religious affiliation, 50% were Muslims and 47.6% were Orthodox Christians (Table 2).

**Table 2:** Socio-demographic characteristics of focus group discussion (FGD) participants in Ethiopia, 2025.

Code	Age	Sex	Marital status	Religion	Educational Status	Role	Region/city administration
FGD1P1	15-19	F	Single	Orthodox	High school	Student	Amhara
FGD1P2	15-19	F	Single	Orthodox	High school	Student	Amhara
FGD1P3	15-19	F	Single	Muslim	High school	Student	Amhara
FGD1P4	15-19	F	Single	Muslim	High school	Student	Amhara
FGD1P5	15-19	F	Single	Muslim	High school	Student	Amhara
FGD1P6	15-19	F	Single	Orthodox	High school	Student	Amhara
FGD1P7	15-19	F	Single	Muslim	High school	Student	Amhara
FGD2P1	15-19	F	Single	Muslim	High school	Student	Afar
FGD2P2	15-19	F	Single	Muslim	High school	Student	Afar
FGD2P3	15-19	F	Single	Orthodox	High school	Student	Afar
FGD2P4	15-19	F	Single	Orthodox	High school	Student	Afar
FGD2P5	15-19	F	Single	Muslim	High school	Student	Afar
FGD2P6	15-19	F	Single	Muslim	High school	Student	Afar
FGD2P7	15-19	F	Single	Orthodox	High school	Student	Afar
FGD3P1	30-39	F	Married	Muslim	College & above	Parent	Addis Ababa
FGD3P2	30-39	F	Married	Orthodox	College & above	Parent	Addis Ababa
FGD3P3	40-49	M	Married	Orthodox	College & above	Parent	Addis Ababa
FGD3P4	40-49	F	Married	Muslim	High school	Parent	Addis Ababa
FGD3P5	30-39	F	Married	Muslim	College & above	Parent	Addis Ababa
FGD3P6	≥50	M	Married	Orthodox	High school	Parent	Addis Ababa
FGD3P7	≥50	M	Married	Orthodox	College & above	Parent	Addis Ababa
FGD4P1	40-49	F	Married	Orthodox	College & above	Parent	Dire Dawa
FGD4P2	40-49	F	Married	Muslim	College & above	Parent	Dire Dawa
FGD4P3	40-49	F	Married	Muslim	High school	Parent	Dire Dawa
FGD4P4	30-39	M	Married	Orthodox	College & above	Parent	Dire Dawa
FGD4P5	40-49	M	Married	Orthodox	College & above	Parent	Dire Dawa
FGD4P6	40-49	F	Married	Muslim	College & above	Parent	Dire Dawa
FGD4P7	40-49	F	Married	Orthodox	High school	Parent	Dire Dawa
FGD5P1	≥50	M	Married	Orthodox	College & above	Religious leader	Amhara
FGD5P2	40-49	M	Married	Orthodox	High school	Religious leader	Amhara
FGD5P3	≥50	M	Married	Muslim	College & above	Religious leader	Amhara
FGD5P4	40-49	M	Married	Muslim	College & above	Religious leader	Amhara
FGD5P5	40-49	M	Married	Protestant	College & above	Religious leader	Amhara
FGD5P6	≥50	M	Married	Orthodox	College & above	Religious leader	Amhara
FGD5P7	≥50	M	Married	Muslim	High school	Religious leader	Amhara
FGD6P1	30-39	F	Married	Muslim	College & above	Community leader	Afar
FGD6P2	30-39	M	Married	Orthodox	College & above	Community leader	Afar
FGD6P3	40-49	M	Married	Muslim	College & above	Community leader	Afar
FGD6P4	≥50	M	Married	Orthodox	High school	Community leader	Afar
FGD6P5	≥50	M	Married	Orthodox	High school	Community leader	Afar
FGD6P6	≥50	F	Married	Muslim	College & above	Community leader	Afar
FGD6P7	40-49	M	Married	Muslim	College & above	Community leader	Afar

NB: To avoid potential risk of participant identification, the serial number is removed; the age, educational status and role are more broadly categorized.

Nineteen key informants were participated in the study. Over half were females (57.9%), most held second degrees (57.9%), and the majority were nurses (63.2%). Their professional experience ranged from 6 to 34 years. The key informants represented four diverse settings—Amhara, Afar, Addis Ababa, and Dire Dawa (Table 3).

**Table 3:** Socio-demographic characteristics of key informant interview participants in Ethiopia, 2025.

Participant code	Sex	Educational status	Profession	Experience (years)	Region/Administrative level
KII_1	M	Second degree	Health Officer	10-20	Federal
KII_2	F	Second degree	Health Officer	10-20	Federal
KII_3	F	First degree	Nurse	<10	Amhara
KII_4	F	First degree	Nurse	<10	Amhara
KII_5	M	Second degree	Nurse	10-20	Addis Ababa
KII_6	F	First degree	Nurse	<10	Dire Dawa
KII_7	M	First degree	Nurse	<10	Amhara
KII_8	F	Second degree	Nurse	>20	Amhara
KII_9	F	Second degree	Nurse	10-20	Afar
KII_10	M	First degree	Medical doctor	10-20	Dire Dawa
KII_11	M	Second degree	Nurse	10-20	Afar
KII_12	M	Second degree	Nurse	10-20	Afar
KII_13	M	Second degree	Health officer	>20	Afar
KII_14	M	Second degree	Nurse	10-20	Amhara
KII_15	F	First degree	Health officer	10-20	Addis Ababa
KII_16	F	Diploma	HEW	10-20	Amhara
KII_17	F	Diploma	HEW	<10	Amhara
KII_18	F	Second degree	Nurse	10-20	Afar
KII_19	F	Second degree	Nurse	10-20	Amhara

NB: HEW = Health extension worker.

To protect participant confidentiality, specific organization names were removed, educational status was more broadly categorized, and exact years of experience have been aggregated into categories.

### Convergent and divergent perspectives across stakeholder groups

Cross-stakeholder analysis identified areas of convergence and divergence in participants’ accounts of HPV vaccination implementation in Ethiopia. Health workers, administrators, parents, adolescent girls, and community and religious leaders commonly recognized the vaccine’s effectiveness in preventing cervical cancer, the operational advantages of school-based delivery, and the potential role of religious endorsements in promoting community acceptance.

However, stakeholders differed in how they perceived and prioritized implementation barriers. Health workers and administrators primarily emphasized structural and logistical challenges, including cold-chain failures, inadequate staffing during school-based campaigns, and coordination gaps among implementing partners. Parents and community representatives more frequently highlighted sociocultural barriers, particularly fertility-related rumours and fears that vaccinating adolescent girls might encourage premarital sexual activity. Religious leaders emphasized moral and theological considerations, while adolescent girls highlighted peer influence as an important factor affecting vaccination decisions.

Contextual differences also emerged in the available accounts. Participants from rural and pastoralist settings described physical inaccessibility and population mobility as challenges to equitable vaccine delivery, whereas participants in urban settings highlighted social media-driven misinformation and resistance to vaccination in some private and international schools. These findings represent patterns observed in participants’ accounts rather than systematic comparisons across geographical settings, as stakeholder groups and locations were not fully cross-represented. Overall, the findings suggest that perceived implementation barriers and enablers vary according to stakeholders’ roles and experiences.

### Implementation enablers and barriers to HPV vaccination

This study identified a wide range of interacting factors, influencing the implementation of HPV vaccination in Ethiopia. The findings were organised into two main overarching categories: enablers that facilitated vaccine uptake and delivery, comprising four categories, and barriers that hindered successful implementation, comprising five categories.

### Primary Enablers of HPV vaccination implementation

#### Category 1: Perceived vaccine attributes and delivery adaptability.

This category captures how participants perceived the inherent attributes of the HPV vaccine and its delivery strategies as enablers of HPV vaccination implementation. Three interrelated subcategories emerged: evidence-based effectiveness, relative advantage, and adaptability of delivery strategies.

Participants described the vaccine’s proven effectiveness in preventing cervical cancer as an important factor influencing its acceptance. They expressed confidence in the evidence supporting HPV vaccine effectiveness and highlighted its ability to prevent cervical cancer as a reason for accepting and supporting HPV vaccination. A key informant noted:

***“HPV vaccination is recognized as the most effective intervention for preventing cervical cancer and saving women’s lives”* (KII-1).**

Participants emphasized the relative advantages of HPV vaccination over cervical cancer screening as factors that enhanced its acceptability. Vaccination was perceived as less invasive, less stigmatizing, and more socially acceptable than cervical cancer screening, which participants associated with discomfort and privacy concerns. Participants also highlighted school-based delivery as convenient for parents and useful for identifying girls eligible for vaccination. One participant explained:

***“The HPV vaccine is easier to administer and more culturally acceptable than screening, which is invasive and exposes private parts.” (FGD2P5)***. A key informant also stated, ***“Girls are easily identified using class rosters, and mothers do not need to leave work to take their adolescent girls to health facilities” (KII-1).***

Participants emphasized the adaptability of delivery strategies as an important facilitator of implementation feasibility. They described programmatic adaptations, including the transition to a single-dose schedule, implementation of multi-age cohort campaigns, and integration of HPV vaccination into routine immunization services as important enablers. Participants perceived these adaptations as reducing operational complexity and resource requirements and improving the feasibility of implementation within existing health system constraints. They also described context-specific delivery approaches, including outreach to pastoralist communities and flexible delivery models in conflict-affected areas, as important for addressing local implementation challenges. A key informant explained:

***“The move to a routinized, single dose and multi-age cohort strategy reduced costs, and made implementation much easier”* (KII-1).** Another added, ***“For hard-to-reach and pastoralist communities, Periodic Intensification of Routine Immunization and similar platforms were used to extend services and ensure coverage. We use a ‘hit and run’ approach in conflict-affected areas”* (KII-2).**

#### Category 2: Supportive policy and multisectoral strategic partnerships.

This category highlights how policy legitimacy, sustained political ownership, and coordinated partnerships collectively created an enabling implementation environment for HPV vaccination. Rather than acting independently, these elements functioned synergistically to institutionalize the programme, mobilize resources, and operationalize delivery. It comprises three interrelated subcategories: political commitment, strategic partnerships, and intersectoral collaboration.

Participants emphasized political commitment as an important facilitator of HPV vaccination implementation. They highlighted the formal inclusion of HPV vaccination in the national routine immunization schedule as evidence of government commitment and described this integration as strengthening government ownership of the programme. Participants also noted the government’s provision of the vaccine free of charge as an important measure supporting vaccination access. A key informant affirmed:

***“The strong political will and inclusion of the HPV vaccine in the routine immunisation schedule is the biggest enabler – ensuring free vaccination and national prioritisation”* (KII-8).** Another added: ***“Ethiopia has signed an agreement to eliminate cervical cancer by 2030”* (KII-2).**

Participants consistently emphasized the importance of sustained support from global partners for HPV vaccination implementation. They identified the Global Alliance for Vaccines and Immunization (GAVI), World Health Organization (WHO), United Nations Children’s Fund (UNICEF), and Bill & Melinda Gates Foundation (BMGF) as key partners providing financial, logistical, procurement, and technical support. Participants described financial assistance as supporting vaccine availability and cold-chain infrastructure, while technical assistance included guidance on implementation protocols, monitoring tools, and training materials. They also emphasized the importance of these contributions in addressing implementation challenges and resource constraints. One key informant explained:

***“Without GAVI and WHO support, we could not run it – they provided vaccines, vaccine carriers, guidelines, everything” (KII-5).***

Participants described strong intersectoral collaboration, particularly between the health and education sectors, as important for reaching the target population. Schools were repeatedly identified as strategic platforms for identifying eligible adolescents, facilitating vaccine delivery, and communicating with parents and caregivers. Participants also described structured coordination processes, including joint planning meetings, shared micro-plans, and clearly defined roles among ministries, local administrators, and partners. These coordination mechanisms were described as facilitating collaboration and addressing implementation challenges. An immunisation expert stated:

***“There is strong inter-sectoral collaboration between the Ministries of Health and Education. We hold regular discussions with stakeholders before each round, which helps us assign roles, solve problems, and coordinate partners effectively”* (KII-2).**

#### Category 3: Socio-cultural and belief-based drivers.

This category illustrates how deeply rooted social norms, belief systems, and lived experiences shaped individual and collective acceptance of HPV vaccination. Rather than acting as passive background factors, socio-cultural influences actively mediated trust, risk perception, and decision-making around vaccination. Three interrelated subcategories emerged: religious endorsement, peer influence, and perceived severity of cervical cancer.

Participants described religious endorsement as an important facilitator of HPV vaccination acceptance and uptake. They highlighted the role of religious leaders as trusted sources of information who could frame HPV vaccination as compatible with religious teachings. Participants reported that religious leaders emphasized principles such as preservation of life, prevention of harm, and acceptance of beneficial health interventions. They also described religious leaders as helping to address moral concerns and misconceptions about HPV vaccination through messages consistent with familiar religious teachings. An Orthodox priest cited 1 Thessalonians 5:21:

***“There is no biblical teaching that prohibits vaccination; rather, the Bible encourages believers to examine new practices carefully with the principle – test everything; hold fast what is good”* (FGD5P2).** A Muslim Ustaz similarly referred to Qur’an 5:32: ***“Vaccination aligns with the Qur’anic command to preserve life – whoever saves one life, it is as if he has saved all of mankind” (FGD5P4).***

Participants described peer influence as an important factor affecting adolescents’ perceptions and acceptance of HPV vaccination. They reported that adolescents’ decisions were influenced by their peers’ experiences and opinions, particularly in school settings. Participants highlighted positive experiences shared by vaccinated girls, including minimal pain and absence of side effects, as helping to reduce fears and address rumours about vaccination. They also reported that such peer experiences encouraged acceptance of HPV vaccination among adolescents who were initially hesitant. A key informant recalled:

**“On the first day of the school campaign, most girls were hesitant, but on the subsequent days, girls who were vaccinated told their friends it was easy, and many more agreed” (KII-1).**

Participants reported that perceptions of cervical cancer severity was an important factor influencing acceptance of HPV vaccination. They commonly described cervical cancer as a fatal and devastating disease and referred to personal or community experiences of illness and death. Participants explained that witnessing the suffering associated with cervical cancer increased their concern about the disease and strengthened their support for HPV vaccination. One mother explained:

***“I know that cervical cancer is a deadly disease and have seen a woman die from it, so we must prevent it”* (FGD4P5).**

#### Category 4: Health system readiness and workforce motivation.

This category describes participants’ perceptions of health system readiness and the role of motivated and proactive frontline health workers in supporting HPV vaccination implementation. Participants described health system readiness not simply in terms of resource availability, but as the alignment of existing infrastructure, coordination mechanisms, and human capacity that they perceived as supporting programme implementation. Three interrelated subcategories emerged: leveraging existing immunisation infrastructure, digital coordination and supervision, and health worker communication and personal testimony.

Participants identified existing immunisation infrastructure as an important facilitator of HPV vaccination implementation. They described the integration of HPV vaccination into the national immunisation programme and the use of established delivery platforms, including cold-chain systems, trained health facility staff, and routine micro-planning practices. Participants reported that incorporating HPV vaccination into existing immunisation workflows alongside other routine childhood immunisation activities reduced logistical challenges and the need for additional resources at subnational and facility levels. A key informant noted:

***“Routinisation of HPV vaccination enables easy implementation using existing health facility staff and cold chain systems”* (KII-18).**

Participants highlighted digital communication and reporting tools as important for programme coordination and supervision. They described the routine use of virtual communication platforms, such as WhatsApp, alongside digital reporting systems such as District Health Information Software 2 (DHIS2), for sharing information across administrative levels. Participants reported that using these tools helped to identify implementation challenges, including supply gaps, low uptake in specific areas, and workforce constraints, and to communicate about responses to these challenges. Respondents also described daily virtual meetings as supporting supervision and accountability during vaccination periods, particularly in geographically dispersed settings. They further reported that digital connectivity facilitated communication among frontline providers, district managers, and regional teams. One respondent stated:

***“Daily virtual meetings and digital tools enabled real-time monitoring, rapid problem-solving, and strong supervision during vaccination”* (KII-2).**

Participants identified health worker communication and personal testimony as important factors influencing HPV vaccine acceptance. They repeatedly highlighted frontline health workers as trusted sources of information within communities. Participants described clear, confident, and empathetic communication by health workers as helping to address parental concerns and misinformation about the HPV vaccine. They also reported that explanations provided during community meetings or facility visits were perceived as more persuasive than messages received through mass media or from external actors. One participant stated:

***“When the nurse stood in front of us and explained clearly, we trusted her more than other sources”* (FGD3P5).**

Beyond technical explanations, participants described personal testimony from health workers, particularly those who had vaccinated their own daughters, as an important source of reassurance and confidence in HPV vaccination. They reported that health workers’ personal experiences and decisions to vaccinate their own daughters strengthened their confidence in the vaccine and encouraged acceptance. One key informant explained:

***“I tell mothers I vaccinated my own daughter, and that reassures them”* (KII-6).**

### Primary barriers to HPV vaccination implementation

Despite numerous enablers, the study identified several barriers at multiple levels of the implementation system.

#### Category 1: Suspicions about targeting 9–14-year-old girls and consent-related administrative obstacles.

This category describes concerns related to the targeting of girls aged 9–14 years and administrative challenges associated with obtaining consent. Two interrelated subcategories emerged: (1) Suspicions about targeting girls aged 9–14 years and (2) Consent-related administrative obstacles.

Participants widely described the vaccination of girls aged 9–14 years as a sensitive issue. They reported that vaccinating girls in this age group generated questions and concerns among some community members about the purpose of the programme. Participants described rumours linking HPV vaccination to fertility control or hidden population-control agendas and reported that such rumours contributed to mistrust of the programme. They also highlighted concerns about limited explanations regarding why HPV vaccination is given before sexual debut, which they perceived as contributing to misunderstanding among parents and caregivers. A focus group participant explained:

***“Rumours were rampant, saying that 14 years is a transition period to reproductive age and was targeted to implement the hidden population control agenda”* (FGD6P7).**

Participants identified parental consent requirements as an important implementation challenge. They described the requirement for written parental consent as difficult to manage in some implementation settings. Participants reported that consent forms were sometimes unreturned, returned late, or submitted with incomplete information. They also described the additional follow-up required from health workers and teachers, including telephone calls and home visits, to obtain or confirm parental approval. A health worker noted:

***“Some girls did not return consent forms, while others returned incomplete forms, requiring follow-up calls to parents to confirm consent, which delayed vaccination”* (KII-8).**

#### Category 2: Pervasive misinformation and cognitive resistance.

This category captures demand-side barriers driven by misinformation, deeply held beliefs, and individual risk perceptions that collectively contributed to resistance toward HPV vaccination. Participants described these barriers as mutually reinforcing, shaping how caregivers, adolescents, and community members interpreted the purpose, safety, and necessity of the vaccine. Three interrelated subcategories emerged: infertility rumours, conspiracy beliefs, and low perceived susceptibility to cervical cancer.

Participants described rumours about infertility as a common source of resistance to HPV vaccination. Many participants reported fears that the HPV vaccine could compromise girls’ future fertility and noted that these concerns were particularly expressed by mothers and caregivers. Participants also described fertility as an important concern when discussing HPV vaccination and reported that rumours and misinformation about infertility circulated through informal social networks and social media platforms. They noted that unverified claims about the vaccine were shared among community members and contributed to concerns about vaccination. A key informant stated:

***“The biggest concern is future fertility… mothers worry this vaccine might prevent their daughters from having children”* (KII-13).**

Participants described conspiracy beliefs as a source of mistrust and resistance to HPV vaccination. Some participants reported concerns that HPV vaccination might encourage early sexual activity by creating a perceived sense of protection or by being interpreted as condoning premarital sex. Participants also reported that these concerns were expressed by some religious leaders and community members and were linked to moral and behavioural concerns about adolescent vaccination. A religious leader observed:

***“People think the vaccine may lead girls to engage in premarital sex”* (FGD5P3).**

Participants also reported conspiracy narratives linking HPV vaccination to hidden global agendas, including references to the Illuminati. They described adolescents as being exposed to similar narratives, particularly through peers and online platforms. An adolescent girl stated:

***“There are rumours about the vaccine, with some saying it is not a vaccine but an Illuminati plot” (FGD1P4).***

Participants described low perceived susceptibility to cervical cancer as a barrier to HPV vaccination, particularly among mothers. Many participants reported that some mothers did not perceive cervical cancer as a personal or immediate threat, partly because they had not observed cases within their families or social circles. Participants also described how the absence of direct experience with cervical cancer was linked with lower concern about the disease and less perceived need for preventive action. Some participants further reported that low concern about cervical cancer occurred alongside misinformation and conspiracy beliefs about HPV vaccination. One mother reflected:

***“I do not know the exact reason… but I perceive that neither I nor my daughter are at risk of cervical cancer… or I believe it cannot happen to us... Unless we see someone close to us suffering from the disease, we tend not to perceive ourselves as being at risk”* (FGD4P5).**

#### Category 3: Health system resource constraints and coordination gaps.

This category illustrates how structural limitations within the health system and weaknesses in coordination across implementing actors undermined the effective delivery of HPV vaccination. Participants consistently described these challenges as interrelated rather than isolated, noting that deficits in infrastructure, human resources, and oversight compounded one another and constrained both the reach and quality of vaccination services. Four interrelated subcategories emerged: cold chain and transport challenges, shortages of well-trained staff, internet access and reporting tool challenges, and limited coordination and supervision.

Participants frequently described cold-chain and transport challenges as barriers to consistent vaccine delivery, particularly in hard-to-reach areas. They reported that ageing refrigeration equipment, unreliable electricity supply, and limited access to transport affected vaccine storage and timely distribution. Participants also described these constraints as limiting outreach activities and, in some cases, resulting in vaccine wastage or missed vaccination sessions. They further reported that such disruptions affected the delivery of vaccination services and created challenges for maintaining reliable vaccination activities at the community level. A health manager stated:

***“Cold chain problems and transport delays often cause missed opportunities for vaccination”* (KII-5).**

Participants highlighted shortages of well-trained staff as an important constraint to HPV vaccination implementation. They reported that school-based vaccination sessions were often managed by only two health workers who were responsible for administering vaccines, providing counselling, completing documentation, and managing potential adverse events. Key informants noted that this workload was particularly burdensome in settings where parental hesitancy was common, as additional time was required to provide explanations and reassurance. A key informant described:

***“Assigning only two health workers per school created excessive workloads, limiting their ability to adequately counsel girls and address concerns – ultimately leading to poor awareness and vaccine hesitancy”* (KII-2).**

Beyond staffing numbers, participants also raised concerns about limited training and counselling capacity among some health workers. They reported that some providers had difficulty responding confidently and consistently to parents’ questions about vaccine safety and eligibility. Participants further described these communication difficulties as affecting parents’ confidence in the information provided by health workers. A parent stated:

***“Some health workers do not have enough training; they struggle to answer questions, making us hesitant to vaccinate our children”* (FGD3P3).**

Participants reported challenges related to internet access and digital reporting tools as barriers to HPV vaccination programme implementation. They described poor or intermittent internet connectivity, particularly in rural and remote areas, as an obstacle to timely data reporting. Participants also described some digital reporting platforms as difficult to use and reported that this contributed to delayed, incomplete, or missing data entry. They further reported difficulties in obtaining timely and complete vaccination information for programme monitoring and follow-up. A key informant stated:

***“Some health workers delay or skip reporting due to poor internet connectivity or because the reporting tools are complicated”* (KII-2).**

Participants reported challenges related to coordination and supervision that affected programme planning and implementation. They described instances in which implementing partners operated independently, resulting in duplication of activities, gaps in responsibility, and confusion about roles at the local level. Participants also reported that, in some instances, schools were informed of vaccination schedules late, leaving insufficient time to mobilise students and engage parents. A key informant reported:

***“Sometimes partners work independently, resulting in overlapping, duplication or missing activities, and creating role confusion”* (KII-3).**

Participants described supervisory support as inconsistent, particularly in remote or insecure woredas. They reported that supervision visits were infrequent and often focused on numerical targets rather than supportive mentoring and quality improvement. Participants also identified resource constraints and security challenges as factors that limited the frequency and scope of supervision of HPV vaccination activities. A participant stated:

***“Due to resource shortages and security problems, we were unable to provide continuous supervision in all woredas, especially in remote or insecure areas”* (KII-7).**

#### Category 4: Donor dependency risks and financial sustainability concerns.

Participants consistently raised concerns about dependence on donor support for HPV vaccination. They acknowledged the contribution of donors to programme initiation, scale-up, and access to HPV vaccination, while expressing concerns about the continuation of programme activities if external support were reduced. Participants also reported uncertainty about the country’s capacity to finance HPV vaccine procurement in the future. The high unit cost of the HPV vaccine was frequently identified as a challenge to increasing domestic financing and government ownership of the programme. Beyond vaccine procurement, participants raised concerns about funding for other programme activities, including cold-chain maintenance, training, supervision, and community engagement. They also reported that donor-funded activities often placed greater emphasis on short-term vaccination coverage targets than on longer-term financing and integration of HPV vaccination into domestic health budgets. A key informant stated:

***“HPV vaccine is costly, and sustaining it without GAVI support would be difficult”* (KII-1).**

#### Category 5: Structural access constraints and equity gaps.

This category explains how structural and contextual inequities limited equitable access to HPV vaccination across different population groups. Two interrelated subcategories emerged: pastoralist lifestyle and displacement, and urban-specific barriers.

Participants consistently described pastoralist mobility and population displacement as challenges to HPV vaccine delivery. In pastoralist communities, they reported that seasonal and livelihood-related mobility made it difficult for health workers to locate and vaccinate eligible girls during scheduled vaccination sessions. Participants noted that families sometimes moved across districts or administrative boundaries, creating challenges for micro-planning, follow-up of missed vaccination opportunities, and estimating the number of eligible girls. Similarly, participants described internally displaced populations as difficult to reach because of instability, temporary settlement arrangements, and limited mechanisms for tracking eligible girls within routine immunisation systems. They reported that these circumstances contributed to missed vaccination opportunities and difficulties in maintaining continuity of vaccination services. Participants also emphasised that existing delivery approaches, which relied heavily on fixed schools and health facilities, were not well suited to the mobility and settlement patterns of pastoralist and displaced populations. A key informant stated:

***“Tracking girls for scheduled vaccination is impossible; families move constantly”* (KII-7).**

Participants described several barriers to HPV vaccination in urban settings. Although urban residents were reported to have relatively better proximity to health facilities and schools, participants identified exposure to vaccine-related misinformation, particularly through social media platforms, as an important concern. They reported that such exposure contributed to fears about the potential effects of HPV vaccination on fertility. Participants also described challenges related to coordination and institutional fragmentation in urban vaccination activities. A key informant explained:

***“In towns, parents are constantly exposed to Facebook, TikTok and YouTube messages claiming the HPV vaccine causes infertility”* (KII-6).**

Participants also highlighted difficulties in engaging private and international schools in HPV vaccination programmes. They reported that these schools were less involved in government-led school health and immunisation activities and described instances in which school administrators questioned the safety or necessity of HPV vaccination. Participants also reported that lengthy approval processes and institutional autonomy contributed to delays in implementing school-based vaccination, with some schools delaying vaccination or opting out of school-based vaccination activities. One key informant described:

***“Private and international schools are difficult to engage – they question government programmes, delay approvals, and sometimes completely opt out of school-based vaccination, leaving many urban girls unvaccinated”* (KII-5).**

## Discussion

This study explored the enablers and barriers affecting the implementation of HPV vaccination in Ethiopia. The enablers included Perceived Vaccine Attributes and Delivery Adaptability, Supportive Policy and Multisectoral Strategic Partnerships, Socio-cultural and Belief-based Drivers, and Health System Readiness and Workforce Motivation. The barriers included Suspicions About Targeting 9–14-year-old Girls and Consent-related Administrative Obstacles, Pervasive Misinformation and Cognitive Resistance, Health System Resource Constraints and Coordination Gaps, Donor Dependency Risks and Financial Sustainability Concerns, and Structural Access Constraints and Equity Gaps.

The perceived evidence-based effectiveness and life-saving potential of HPV vaccination may represent important enablers of programme implementation, consistent with previous studies [28,29]. Confidence in the scientific evidence supporting vaccine effectiveness may strengthen perceived benefits of vaccination and the perceived value of preventing cervical cancer and related mortality. This interpretation is consistent with the Health Belief Model (HBM), particularly the constructs of perceived benefits, perceived severity, and cues to action. Perceiving HPV vaccination as effective may strengthen its perceived benefits, while recognising cervical cancer as a serious and potentially fatal disease may increase the perceived value of preventive action. Evidence-based information may also serve as a cue to action by reinforcing confidence in vaccination decisions [30]. However, the potential benefits of these positive perceptions may be undermined by misinformation, including infertility rumours and conspiracy beliefs, as well as low perceived susceptibility to cervical cancer [31–34]. These findings suggest that providing information about vaccine effectiveness alone may not be sufficient to address hesitancy. Communication strategies therefore need to combine evidence-based information with trust-building approaches that are culturally sensitive and responsive to locally circulating concerns.

School-based delivery represents an important facilitator of HPV vaccination implementation. Schools offer an efficient platform to reach large numbers of eligible girls before sexual debut and are particularly important in Ethiopia, where primary schools are widely distributed, including in remote areas [35,36]. The potential value of school-based delivery is consistent with Ethiopia’s and WHO’s cervical cancer elimination strategies, which identify schools as important platforms for HPV vaccine delivery [35,37]. However, the findings also suggest that the effectiveness of school-based delivery depends on appropriate preparation and supportive implementation. Written parental consent requirements may create operational challenges and delay vaccination activities [38,39]. Moreover, reliance on schools as primary delivery points may leave out-of-school girls underserved [31,39–42]. These findings highlight the need to strengthen school-based delivery through adequate preparation and coordination, while exploring feasible and ethically appropriate consent mechanisms. Complementary delivery approaches tailored to out-of-school girls are also needed to reduce gaps in access and ensure that school-based strategies do not inadvertently widen vaccination inequities.

This study showed HPV vaccination as a more feasible cervical cancer prevention strategy than screening. This aligns with Ethiopia’s National Cancer Control Plan which designates vaccination as the primary strategy and screening as a secondary priority, with only 8.4% of eligible women screened [35,36]. This is also consistent with the global cervical cancer elimination strategy, which recognises vaccination as the most effective intervention if delivered before HPV exposure [29,43]. The implication is not that screening should be abandoned – it remains essential for those already beyond the vaccination window – but that vaccination must be prioritised as the most efficient, equitable, and forward-looking cervical cancer elimination strategy, particularly in resource-limited settings like Ethiopia.

Our findings point out the importance of recent policy shifts to a single-dose schedule, multi-age cohort (MAC) campaigns, and the routinisation of HPV vaccination. These pragmatic adaptations reduced programme complexity and operational costs, supporting long-term sustainability [44]. Evidence indicates that single-dose schedules provide comparable protection to multi-dose regimens and have been endorsed by WHO’s Strategic Advisory Group of Experts as cost-effective [45]. The implementation of the MAC campaign, which reached more than 6.8 million girls aged 9–14 years in a single year, further demonstrates the potential of age-expansion strategies to accelerate population coverage [17]. Routinisation may also support sustainability by embedding HPV vaccination within existing immunisation systems rather than maintaining parallel delivery structures [46, 47]. However, long-term sustainability will also depend on adequate financing, workforce capacity, supply systems, and effective implementation at subnational levels. The findings underscore the need for maintaining these policy adaptations while addressing the broader resource and implementation constraints identified in this study for sustaining HPV vaccination in Ethiopia.

The current study found that supportive national policies and multisectoral partnerships were essential enablers of HPV vaccination implementation. Strong political commitment, reflected in leadership prioritisation, policy alignment, and cross-sector collaboration – particularly between the health and education sectors – enabled effective programme delivery [48]. Ethiopia’s experience mirrors that of Rwanda, where high coverage was achieved through sustained political leadership and coordinated action [49]. However, reliance on donor support raised concerns about long-term sustainability as Ethiopia approaches graduation. Similar challenges have been reported in other transitioning countries [50,51]. Without clear strategies for domestic resource mobilisation, HPV vaccination coverage may decline. These findings highlight the need to engage ministries of finance and health, explore domestic financing mechanisms, and institutionalise partnerships to ensure continuity beyond donor support.

Our study identified favourable socio-cultural and belief-based perceptions, particularly religious endorsement, positive peer influence, and recognition of cervical cancer severity, as enablers of HPV vaccination. This reflects the pivotal role of religious leaders in countering misinformation and building trust, as well as the influence of significant others in normalising vaccination through shared experiences [52,53]. Perceived severity, often shaped by witnessing illness or death, further motivates caregivers by making cervical cancer risks tangible rather than abstract [54]. Conversely, misinformation – particularly fears of infertility and suspicions surrounding vaccinating girls aged 9–14 years – remained widespread and deeply entrenched. The belief that the HPV vaccine represents a “Western” or “Illuminati” plot to control African populations, together with concerns that it may promote sexual promiscuity, were contributing to vaccine hesitancy, consistent with findings from other African contexts [32–34,55]. Additionally, low perceived susceptibility due to lack of direct experience created an “optimism bias”, reducing perceived risk [52]. These findings highlight the importance of culturally tailored communication strategies that engage trusted community figures, address emotional and moral concerns, and use lived experiences to make cervical cancer risk personally relevant.

This study showed that health system readiness and workforce motivation played a central role in HPV vaccination implementation. As front-line health workers are widely trusted and serve as critical intermediaries between the health system and communities, their effective interpersonal communication, culturally appropriate messaging, and sharing of personal testimonies build trust that no mass media campaign can achieve [35,56]. The use of digital tools such as WhatsApp and DHIS2 further supported implementation by enabling coordination, supervision, and real-time problem-solving, helping to address bottlenecks before they affected coverage [57,58]. However, other health system constraints such as cold chain limitations, unreliable power supply, and ageing equipment restricted outreach capacity and contributed to vaccine wastage, especially in remote areas [59,60]. Shortages of well-trained staff during school-based campaigns also increased workloads, limited meaningful interaction and effective communication with clients, and contributed to vaccine hesitancy as clients’ questions remained unanswered [61]. In addition, poor internet connectivity, complex reporting systems, coordination gaps among implementing partners, and infrequent supervision in remote or insecure settings limited implementation quality and reach [62–65]. These findings underscore the need for adequate staffing with proper training, simplified and offline-capable digital tools, and supportive supervision that strengthens both coverage and quality.

This study revealed structural access and equity gaps in HPV vaccination, disproportionately affecting pastoralist communities, displaced populations, and urban girls in private and international schools. Pastoralists frequent mobility made tracking eligible girls difficult, while displaced populations were often excluded from routine data systems and vaccination activities [24]. Importantly, these findings provide qualitative context for the unexpected finding from our parent study, in which urban residence was associated with HPV vaccine non-uptake [23]. Despite better health infrastructure, urban settings presented distinct challenges, including increased exposure to anti-vaccine misinformation on social media and parental unavailability due to work commitments, which led to delayed or missed consent and exclusion of girls from scheduled vaccination [40,41,66]. In addition, reluctance among private and international schools to participate in public vaccination programmes resulted in substantial numbers of urban girls remaining unvaccinated [42]. Thus, the apparent urban advantage in physical access to health services may not necessarily translate into effective access to HPV vaccination, as urban girls may experience distinct social, informational, institutional, and consent-related barriers. These equity challenges underscore the need to design tailored strategies: mobile outreach for pastoralists, inclusion of displaced populations in humanitarian responses, and targeted engagement with urban private schools.

### Strengths and limitations

This study has several strengths. The use of CFIR 2.0 to develop interview guides and codes enhanced study rigour by ensuring comprehensive inclusion of multilevel implementation factors and improving the transferability of findings. Additionally, incorporating diverse perspectives from health administrators in governmental and non-governmental organisations, frontline health workers, community representatives, parents, and adolescent girls strengthened the study’s ability to capture multi-level influences throughout the system. Moreover, purposive sampling across four diverse settings enabled the study to capture a range of contextual perspectives and implementation experiences, thereby enhancing the contextual richness and transferability of the findings across different settings.

However, the following limitations should be considered when interpreting these findings. First, although participants were informed that all data would be anonymised and used solely for research purposes, social-desirability bias may still have influenced responses, particularly on sensitive issues such as sexuality, religious beliefs, and government programmes. This may have led to underreporting of stigmatised views or concerns and overreporting of socially acceptable attitudes. Second, the adolescent FGD participants were exclusively in-school girls aged 17–18 years and were selected partly based on academic engagement and involvement in school activities. This sampling approach may have favoured more informed, engaged, or articulate adolescents and may therefore have underrepresented barriers experienced by less-engaged girls and those with limited access to schooling. The participants were also older than the current HPV vaccination target group of 9–14 years; consequently, their experiences may not fully reflect the developmental, parental, and decision-making circumstances of the current target cohort, potentially underestimating the influence of parental decision-making and age-specific barriers among younger girls. Similarly, out-of-school girls were not directly represented, although these groups may face distinct access and implementation barriers; their exclusion may therefore have underestimated barriers among harder-to-reach populations. Third, the composition of FGDs 3, 4.5 and 6, including mixed-gender groups and participants from different religious backgrounds, may have constrained discussion of sensitive issues and contributed to underreporting of concerns related to sexuality, fertility, or vaccination. Fourth, this qualitative analysis explored stakeholders’ perceived barriers and enablers but did not formally quantify or rank their relative importance. Finally, interviews conducted in Amharic and translated into English may have introduced translation or interpretation loss, particularly for culturally specific expressions, or meanings. Although transcripts were checked against recordings and field notes, some contextual meanings may not have been fully retained. In Afar, recruitment was restricted to Amharic-speaking bilingual individuals, potentially excluding non-Amharic-speakers, limiting the diversity of sociocultural perspectives.

## Conclusions and recommendations

### Conclusions

The implementation of HPV vaccination in Ethiopia has shown substantial progress, driven by Perceived Vaccine Attributes and Delivery Adaptability, Supportive Policy and Multisectoral Strategic Partnerships, Socio-cultural and Belief-based Drivers, and Health System Readiness and Workforce Motivation. However, Suspicions About Targeting 9–14-year-old Girls and Consent-related Administrative Obstacles, Pervasive Misinformation and Cognitive Resistance, Health System Resource Constraints and Coordination Gaps, Donor Dependency Risks and Financial Sustainability Concerns, and Structural Access Constraints and Equity Gaps remain critical constraints. The implication is that sustaining HPV vaccination in Ethiopia requires a dual strategy: maintaining and reinforcing the existing enablers (policy, partnerships, community trust) while systematically dismantling the barriers through equity-focused investments, domestic financing commitments, and sustained community engagement.

### Recommendations

Evidence-informed priorities for strengthening HPV vaccination implementation include:

1. Strengthen culturally tailored communication via trusted community, health-worker, and digital channels to counter misinformation, infertility fears, and conspiracy beliefs.
2. Strengthen engagement with religious and community leaders as trusted sources of information and dialogue to address sociocultural concerns and improve community understanding and acceptance of HPV vaccination.
3. Review and simplify consent and administrative procedures for school-based HPV vaccination to reduce avoidable delays while maintaining ethical standards.
4. Strengthen health-system readiness for school-based vaccination by addressing health-worker workload, staffing, training, cold-chain capacity, and coordination challenges.
5. Strengthen the sustainability of HPV vaccination financing by progressively exploring increased domestic resource mobilization and longer-term financing arrangements alongside continued engagement with external partners.
6. Strengthen supportive supervision and coordination by emphasizing problem-solving, quality improvement, timely feedback, and appropriate use of digital approaches, particularly where geographical access to schools and health facilities is challenging.
7. Improve the inclusion of underserved settings and populations in vaccination planning, including private and international schools in urban areas and pastoralist and conflict-affected populations, through context-specific delivery and communication strategies.
8. Improve the usability and adaptability of digital monitoring and reporting tools, particularly in settings with limited connectivity, while strengthening mechanisms for identifying and responding to local implementation barriers.
9. Conduct implementation and operational research to evaluate alternative consent mechanisms, mobile delivery models, communication interventions, private-school engagement, and strategies for reaching out-of-school girls and mobile populations.

## Supporting information

S1 File Summary of qualitative findings on enablers and barriers to HPV vaccination implementation in Ethiopia. This supplementary table presents the qualitative analysis of key informant interviews, and focus group discussions, including verbatim meaning units, condensed meaning units, CFIR 2.0 codes, subcategories, and main categories describing the enablers and barriers to HPV vaccination implementation.
(DOCX)

S2 File Operationalization of CFIR 2.0 domains and constructs for the analysis of HPV vaccination implementation in Ethiopia. This table provides the operational definitions assigned to CFIR 2.0 constructs, categorised across the five domains: Innovation, Outer Setting, Inner Setting, Individuals, and Implementation Process. These definitions were contextualised to the Ethiopian HPV vaccination programme to ensure transparent, consistent, and reproducible application of the framework during deductive coding of key informant interviews and focus group discussions. The operationalisation clarifies how each construct was interpreted within the local implementation landscape, guiding data categorisation and interpretation.
(DOCX)

S3 File Consolidated criteria for reporting qualitative studies (COREQ): 32-item checklist. This supplementary file presents the completed COREQ checklist, documenting adherence to qualitative research reporting standards across three domains: research team and reflexivity, study design, and analysis and findings. It includes participant selection, data collection, coding, theme development, and quotation presentation, thereby supporting the transparency, rigour, and credibility of the study.
(PDF)
