# Supplementary material for: Enablers and barriers to human papillomavirus vaccination implementation among key stakeholders in Ethiopia: A qualitative study
Source: PLoS One. 2026 Nov 2;21(11):e0361925. doi: 10.1371/journal.pone.0361925 (PMC13726970; doi:10.1371/journal.pone.0361925)
Supplement: Supplementary file 1 [file pone.0361925.s003.pdf]

Consolidated criteria for reporting qualitative studies (COREQ): 32-item checklist

Please indicate in which section each item has been reported in your manuscript. If you do not feel an item applies to your manuscript, please enter N/A.

For further information about the COREQ guidelines, please see Tong *et al.*, 2017: https://doi.org/10.1093/intqhc/mzm042

No.	Item	Description	Section #
**Domain 1: Research team and reflexivity**			
Personal characteristics			
1.	Interviewer/facilitator	Which author/s conducted the interview or focus group?	ıcted KIIs; PI and
2.	Credentials	What were the researcher's credentials? *E.g. PhD, MD*	and KA are Senic
3.	Occupation	What was their occupation at the time of the study?	Systems Strategi
4.	Gender	Was the researcher male or female?	:tion Procedures: .
5.	Experience and training	What experience or training did the researcher have?	ining in Reproduc
Relationship with participants			
6.	Relationship established	Was a relationship established prior to study commencement?	h relevant stakeh
7.	Participant knowledge of the interviewer	What did the participants know about the researcher? *E.g. Personal goals, reasons for doing the research*	ıced to PI as a res
8.	Interviewer characteristics	What characteristics were reported about the interviewer/facilitator? *E.g. Bias, assumptions, reasons and interests in the research topic*	vaccination, poten
**Domain 2: Study design**			
Theoretical framework			
9.	Methodological orientation and theory	What methodological orientation was stated to underpin the study? *E.g. grounded theory, discourse analysis, ethnography, phenomenology, content analysis*	constructivist and
Participant selection			
10.	Sampling	How were participants selected? *E.g. purposive, convenience, consecutive, snowball*	eneous purposive
11.	Method of approach	How were participants approached? *E.g. face-to-face, telephone, mail, email*	y representatives (
12.	Sample size	How many participants were in the study?	ınd Sample Size: S
13.	Non-participation	How many people refused to participate or dropped out? What were the reasons for this?	vith the planned FC
Setting			
14.	Setting of data collection	Where was the data collected? *E.g. home, clinic, workplace*	described, includir
15.	Presence of non-participants	Was anyone else present besides the participants and researchers?	: Moderators/interv

16.	Description of sample	What are the important characteristics of the sample? *E.g. demographic data, date*	tics are provided in
Data collection			
17.	Interview guide	Were questions, prompts, guides provided by the authors? Was it pilot tested?	ıre/previous tools a
18.	Repeat interviews	Were repeat interviews carried out? If yes, how many?	t interviews were c
19.	Audio/visual recording	Did the research use audio or visual recording to collect the data?	es: FGDs and KIIs
20.	Field notes	Were field notes made during and/or after the interview or focus group?	; during FGDs; fiel
21.	Duration	What was the duration of the interviews or focus group?	FGDs lasted 60–1
22.	Data saturation	Was data saturation discussed?	ely for FGDs and K
23.	Transcripts returned	Were transcripts returned to participants for comment and/or correction?	ı their accuracy an
**Domain 3: analysis and findings**			
Data analysis			
24.	Number of data coders	How many data coders coded the data?	ıct definitions and
25.	Description of the coding tree	Did authors provide a description of the coding tree?	meaning units → c
26.	Derivation of themes	Were themes identified in advance or derived from the data?	analysis: CFIR 2.0
27.	Software	What software, if applicable, was used to manage the data?	was used for data r
28.	Participant checking	Did participants provide feedback on the findings?	pretations were sha
Reporting			
29.	Quotations presented	Were participant quotations presented to illustrate the themes / findings? Was each quotation identified? *E.g. Participant number*	ıs verbatim quotati
30.	Data and findings consistent	Was there consistency between the data presented and the findings?	ıdings are supporte
31.	Clarity of major themes	Were major themes clearly presented in the findings?	enabler categorie
32.	Clarity of minor themes	Is there a description of diverse cases or discussion of minor themes?	presented under ea

When submitting your manuscript via the online submission form, please upload the completed checklist as a Figure/supplementary file.

If you would like this checklist to be included alongside your article, we ask that you upload the completed checklist to an online repository and include the guideline type, name of the repository, DOI and license in the *Data availability* section of your manuscript.

Developed from: Allison Tong, Peter Sainsbury, Jonathan Craig, Consolidated criteria for reporting qualitative research (COREQ): a 32-item checklist for interviews and focus groups, International Journal for Quality in Health Care, Volume 19, Issue 6, December 2007, Pages 349–357, https://doi.org/10.1093/intqhc/mzm042
