# Supplementary material for: Enablers and barriers to human papillomavirus vaccination implementation among key stakeholders in Ethiopia: A qualitative study
Source: PLoS One. 2026 Nov 2;21(11):e0361925. doi: 10.1371/journal.pone.0361925 (PMC13726970; doi:10.1371/journal.pone.0361925)
Supplement: Supplementary file 2 [file pone.0361925.s001.docx]

**Supporting file 1. Summary of qualitative findings on enablers and barriers to HPV vaccination implementation in Ethiopia.**

**Enablers to HPV Vaccination Implementation**

| **Meaning unit (Verbatim quote)** | **Condensed meaning unit** | **Code/s** | **Subcategory** | **Main category** |
| --- | --- | --- | --- | --- |
| “HPV vaccination is recognized as the most effective intervention for preventing cervical cancer and save women's lives.” (KII1) | HPV vaccine recognised as most effective for cervical cancer prevention. | - Perceived effectiveness | Evidence-based effectiveness | 1. **Perceived Vaccine Attributes and Delivery Adaptability** |
| “School-based delivery helps easy identification of girls using class rosters, and mothers do not need to leave work to go to health facilities.” (KII1) | School delivery simplifies identification of girls and avoids work disruption. | - Easy identification of girls - Reduced opportunity cost for mothers | Relative advantage |  |
| “The HPV vaccine is easier to administer and more culturally acceptable than screening, which is invasive and exposes private parts.” (FGD2P5) | Vaccine preferred over invasive screening that exposes private parts. | - Easy to administer - Less invasive - Preserves privacy |  |  |
| “The move to a single dose and multi age cohort campaign reduced costs and made implementation much easier. Now that HPV is part of routine immunisation, it can be given like any other vaccine without extra campaigns.” (KII1) | Single‑dose, multi‑age, and routinisation reduced cost and complexity. | - Shift to single‑dose schedule - Shift to multi‑age cohort - Routinising the vaccine | Adaptability |  |
| “The strong political will and inclusion of HPV vaccine in the national routine immunization schedule is the biggest enabler –ensuring free vaccination and national prioritization.” (KII8) | Political will and national routine schedule ensure free vaccination. | - Political commitment - Routinizing the vaccine - Free vaccination - National prioritization | National political commitment | 2. **Supportive Policy and Strategic Partnerships** |
| “Ethiopia has signed an agreement to eliminate cervical cancer by 2030 and is currently implementing it.” (KII2) | Ethiopia committed to global cervical cancer elimination by 2030. | - Ethiopia’s commitment to eliminate cervical cancer |  |  |
| “Without GAVI and WHO support, we could not run it – they provided vaccines, vaccine carriers, guidelines, everything.” (KII5) | Challenging to cascade the program without support. | - Donor support | Global and external partnerships |  |
| “There is strong inter-sectoral collaboration between the Ministries of Health, and education… In-schools it is easy to access girls and to communicate parents for consent.” (KII2) | Health‑education collaboration facilitates implementation. | - Intersectoral collaboration | Intersectoral collaboration |  |
| “There is no biblical teaching that prohibits vaccination; rather, the Bible encourages believers to examine new practices carefully with the principle – test everything; hold fast what is good (1 Thessalonians 5:21).” (FGD3P2) | No biblical prohibition of vaccination; Bible encourages examining new practices. | - Biblical endorsement - Scriptural openness to innovation | Religious endorsement of vaccination | 3. **Socio-cultural and Belief-based Drivers** |
| “Vaccination aligns with the Qur’anic command to preserve life – ‘whoever saves one life, it is as if he has saved all of mankind’ (Qur’an 5:32).” (FGD3P4) | Vaccination aligns with Qur’anic command to preserve life. | - Qur'anic endorsement - Life preservation mandate |  |  |
| “On the first day of the school campaign many girls were hesitant…but on the subsequent days, girls who were vaccinated told their friends it was easy – and many more agreed.” (KII1) | Positive influence of peers encourages vaccination. | - Peer testimonies | Positive peer influence |  |
| “I know that cervical cancer is a deadly disease and have seen a woman die from it … so we must prevent it.” (FGD2P5) | Personal experience on severity of cervical cancer motivates prevention. | - Perceived severity of disease - Lived experience | Perceived severity of cervical cancer |  |
| “Full routinization of HPV vaccination would enable easy implementation using existing health facility staff and cold chain systems.” (KII3) | Routinisation would allow use of existing staff and cold chain. | - Use of existing health system capacity | Existing immunization infrastructure | 4. **Health System Readiness and Workforce Motivation** |
| “Daily virtual check-ins with the regional and woreda teams using digital platforms such as What is up enabled problem solving.” (KII2) | Routine communication enabled problem‑solving. | - Digital coordination mechanisms - Rapid problem‑solving | Digital coordination and supervision |  |
| “When the nurse explained clearly, we trusted her more than rumours.” (FGD2P5) | Providers’ clear explanation built more trust. | - Health worker communication - Building trust | Health worker communication and personal testimony |  |
| “I tell mothers I vaccinated my own daughter, and that reassures them.” (KII6) | Health workers use of the service reassures mothers. | - Provider personal testimony - Role modelling reassures mothers |  |  |
| “Before the vaccination starts, we always conduct micro-planning from the kebele level upward. This helps us estimate the number of eligible girls and prepare logistics ahead of time.” (KII1) | Bottom‑up micro‑planning helps to estimate eligible girls and logistics. | - Bottom-up micro-planning - Estimate eligible girls - Prepare logistics | Micro-planning and preparation |  |
| “We hold regular discussions with multiple stakeholders before each round. These meetings help us assign roles, solve problems, and coordinate effectively with all partners.” (KII2) | Regular multi‑stakeholder meetings help to effectively coordinate partners. | - Multi‑stakeholder engagement | Multi-stakeholder engagement |  |
| “For hard-to-reach and pastoralist communities, Periodic Intensification of Routine Immunization (PIRI) and similar platforms were used to extend services and ensure coverage... In areas affected by active conflict such as Amhara region, we use a 'hit-and-run' approach.” (KII2) | For pastoralist and conflict‑affected areas, PIRI and “hit‑and‑run” approaches tailored to context. | - PIRI platform for pastoralist communities - Hit‑and‑run strategy for conflict affected areas | Context-tailored delivery strategies |  |
| “Daily virtual meetings and digital tools (dashboards and ODK) enabled real-time monitoring, rapid problem-solving, and supervision during vaccination.” (KII2) | Daily virtual meetings enabled real‑time monitoring and feedback. | - Virtual meetings - Digital tools - Problem solving - Real time monitoring and supervision | Digital coordination and adaptive monitoring/supervision |  |

**Barriers to HPV Vaccination Implementation**

| **Meaning unit (Verbatim quote)** | **Condensed meaning unit** | **CFIR 2.0 Code** | **Subcategory** | **Main category** |
| --- | --- | --- | --- | --- |
| “14 years is a transition period to reproductive age and a sensitive age group. So, rumours were rampant, saying that this age group was targeted to implement the hidden population control agenda.” (FGD2P7) | Targeting adolescents become source of rumours for hidden population control agenda. | - Sensitive age group - Population control conspiracy | Sensitivity of adolescent target group | 1. **Suspicions about Targeting 9–14-year-old girls and Consent-related Administrative Obstacles** |
| “Some girls didn’t return consent forms at all while others return incomplete forms, requiring follow-up calls to parents to confirm consent which delay vaccination.” (KII8) | Missing or incomplete consent forms delay vaccination. | - No return of consent forms - Return of incomplete consent forms - Delays due to consent verification | Complexity of consent procedures |  |
| “Unlike infant vaccines delivered at health facilities, HPV vaccination requires adolescents to be mobilized either through schools or community outreach.” (KII1) | HPV vaccination requires school/community mobilisation. | - Campaign‑based delivery - Community/school mobilization | Challenges of campaign-based delivery |  |
| “The biggest concern is future fertility… mothers worry this vaccine might prevent their daughters from having children.” (KII13) | Risk of infertility is the biggest concern. | - Future infertility rumours | Infertility rumours | 2. **Pervasive Misinformation and Cognitive Resistance** |
| “People think the vaccine may lead girls to engage in premarital sex.” (FGD3P3) | Belief that vaccine leads to premarital sex. | - Encourage premarital sex | Promiscuity concerns |  |
| “There are rumours about the vaccine, with some saying it’s not a vaccine but an Illuminati plot.” (FGD1P4) | Rumours of Illuminati plot, not a real vaccine. | - Conspiracy beliefs - Illuminati narrative | Conspiracy beliefs (Illuminati) |  |
| “Cold chain problems and transport delays often cause missed opportunities for vaccination.” (KII5) | Cold chain and transport delays cause missed vaccination. | - Cold chain failures - Transport delays - Missed opportunities | Cold chain and transport challenges | 3. **Health System Resource Constraints and Coordination Gaps** |
| “Assignment of only two health workers per school created excessive workloads, including vaccination, counselling, recording, and management of adverse events, limiting their ability to adequately counsel recipients and address concerns, which ultimately leading to poor awareness and vaccine hesitancy.” (KII2) | Deploying inadequate health workers per school create excessive overload, resulting in poor counselling and hesitancy. | - Staff shortage - Excessive workload - Poor counselling - Hesitancy spillover | Excessive workload-and poor service quality |  |
| “Some health workers don’t have enough training due to reduced number of training days; they struggle to answer questions, making us hesitant to vaccinate our children.” (FGD2P3) | Inadequate training limited health workers ability to answer questions which leads to parental hesitancy. | - Inadequate training - Inability to address concerns | Inadequate training and counselling capacity |  |
| “Sometimes partners work independently resulting in overlapping or duplication of activities and create role confusion.” (KII3) | Poor partner coordination results overlapping of activities. | - Partner fragmentation - Overlapping activities - Role confusion | Poor partner coordination and role confusion |  |
| “Sometimes the schools are informed late, so when we arrive for vaccination, the girls are not prepared.” (KII) | Delayed school communication affect implementation. | - Late school notification - Unprepared girls |  |  |
| “Due to resource shortages and security problems, we were unable to provide continuous supervision in all woredas, especially in remote or insecure areas.” (KII7) | Resource and security gaps prevent supervision in remote/insecure areas. | - Supervision gaps - Resource shortages - Security constraints | Inadequate supervision due to resource and security constraints |  |
| “Some health workers delay or skip reporting due to poor internet connectivity or the reporting tools are complicated.” (KII2) | Poor internet or complicated tools cause reporting delays/skips. | - Reporting obstacles - Connectivity issues - Complex tools | Reporting delays and data challenges |  |
| “HPV vaccine is very expensive, and sustaining it without Gavi support would be very difficult.” (KII1) | Vaccine is expensive and sustainability difficult without support. | - High vaccine cost - Sustainability risk | Donor dependency | 4. **Donor Dependency Risks and Financial Sustainability Concerns** |
| “In pastoralist areas, tracking girls for second doses is nearly impossible; families move constantly.” (KII7) | Accessing girls is challenging in pastoralist communities. | - Difficulty of tracking girls - Pastoralist mobility | Pastoralist mobility and displacement | 5. **Structural Access Constraints and Equity Gaps** |
| “In towns, parents are constantly exposed to Facebook, TikTok, and YouTube messages claiming the HPV vaccine causes infertility.” (KII6) | Urban parents are exposed to social media misinformation. | - Social media misinformation exposure in urban areas | Social media misinformation exposure |  |
| “Many urban parents support vaccination in principle, but they are busy and miss school meetings. Consent forms are not returned on time, so girls are excluded on vaccination day.” (KII1) | Busy urban parents miss meetings, which delay consent and exclude girls. | - Parental unavailability - Missed school meetings - Delayed consent return | Parental unavailability and consent delays |  |
| “Private and international schools are difficult to engage – they question government programs, delay approvals, and sometimes completely opt out of school-based vaccination, leaving many urban girls unreached.” (KII5) | Delays or opt‑outs by private/international schools from government programs leave urban girls unvaccinated. | - Private school reluctance to participate in government programs - Approval delays | Private and international school opt-out |  |
