# Supplementary material for: Enablers and barriers to human papillomavirus vaccination implementation among key stakeholders in Ethiopia: A qualitative study
Source: PLoS One. 2026 Nov 2;21(11):e0361925. doi: 10.1371/journal.pone.0361925 (PMC13726970; doi:10.1371/journal.pone.0361925)
Supplement: Supplementary file 3 [file pone.0361925.s002.docx]

**Supporting file 2. Operationalization of CFIR 2.0 domains and constructs for the analysis of HPV vaccination implementation in Ethiopia.**

| CFIR 2.0 domain | CFIR 2.0 construct | Operational definition as applied in this study |
| --- | --- | --- |
| **Innovation** | Evidence base | Stakeholders’ perceptions of the scientific credibility, proven effectiveness, and life‑saving value of HPV vaccination in preventing cervical cancer |
|  | Relative advantage | Perceived benefits of HPV vaccination compared with alternative prevention strategies (eg, screening) and advantages of school‑based delivery compared with facility‑based approaches |
|  | Adaptability | Extent to which the HPV vaccination program could be modified to fit the Ethiopian context, including transition to a single‑dose schedule, use of multi‑age cohort campaigns, and integration into routine immunization |
|  | Design and complexity | Perceived difficulty of implementing HPV vaccination, including sensitivity of the target age group, consent procedures, and delivery strategies |
| **Outer setting** | Policies and laws | National political commitment, policy alignment with cervical cancer elimination goals, and inclusion of HPV vaccination in the routine immunization schedule |
|  | Partnerships and connections | Engagement and support from external partners (e.g., Gavi, WHO, UNICEF) through provision of vaccines, funding, technical assistance, and logistics |
|  | Local attitudes and societal pressure | Community norms, cultural and religious beliefs, peer influence, rumors, misinformation, and social media narratives shaping HPV vaccine acceptance |
|  | Financing | Availability and sustainability of funding for HPV vaccination, including reliance on donor support for vaccine procurement and program operations |
|  | Local conditions | Contextual factors such as population mobility, displacement, insecurity, and pastoralist lifestyles that influence access to and continuity of vaccination services |
| **Inner setting** | Available resources | Availability and adequacy of vaccines, cold chain equipment, transport, infrastructure, staffing, and operational funds |
|  | Compatibility | Fit of HPV vaccination within existing EPI structures, workflows, and service delivery platforms |
|  | Communications | Quality and timeliness of formal and informal information exchange across levels of the health system |
|  | Leadership engagement | Commitment and involvement of facility, district, regional, and national leaders in planning, oversight, and problem‑solving |
| **Individuals** | Innovation deliverers – capability | Health workers’ knowledge, skills, and confidence to deliver HPV vaccination and respond to caregiver concerns |
|  | Innovation deliverers – motivation | Health workers’ attitudes, personal endorsement, and commitment to promoting HPV vaccination |
|  | Innovation recipients – need | Caregivers’ and adolescents’ perceptions of cervical cancer severity and the necessity of HPV vaccination |
|  | Innovation recipients – motivation | Willingness of parents and adolescents to accept HPV vaccination, shaped by trust, fear of side effects, and perceived susceptibility |
| **Implementation process** | Planning | Use of structured micro‑planning, bottom‑up headcounts, school rosters, and identification of in‑school and out‑of‑school girls |
|  | Engaging and teaming | Involvement of multiple stakeholders, including teachers, school leaders, religious leaders, community volunteers, and implementing partners |
|  | Doing | Execution of HPV vaccination activities through school‑based delivery, outreach, campaigns, and routine services |
|  | Tailoring strategies | Adaptation of delivery approaches to specific contexts, such as pastoralist, urban, or conflict‑affected settings |
|  | Reflecting and evaluating | Monitoring, supervision, reporting, feedback, and use of routine data systems (eg, DHIS2, ODK) to assess implementation performance |
