# Supplementary material for: Enablers and barriers to human papillomavirus vaccination implementation among key stakeholders in Ethiopia: A qualitative study
Source: PLoS One. 2026 Nov 2;21(11):e0361925. doi: 10.1371/journal.pone.0361925 (PMC13726970; doi:10.1371/journal.pone.0361925)
Supplement: Supplementary file 4 [file pone.0361925.s005.docx]

**Point-by-Point Response to Editor and Reviewers**

**Manuscript Title:** **Enablers and Barriers to Human Papillomavirus Vaccination Implementation among key stakeholders in Ethiopia: A Qualitative Study**

We sincerely thank the Editor and all reviewers for their constructive and insightful comments. Below we provide a comprehensive point-by-point response to all comments and suggestions. We have carefully addressed each comment and believe the revisions have substantially strengthened the manuscript. All changes have been highlighted in the revised manuscript, with page and line numbers indicated for each revision.

**Response to Editor**

| **Sub-topic** | **Comments / Suggestions Raised** | **Response / Justification** | **Page & Line Numbers** |
| --- | --- | --- | --- |
| **Style Requirements** | 1. Please ensure that your manuscript meets PLOS ONE's style requirements, including those for file naming. | We sincerely thank the editor for this important reminder. We have carefully reviewed the PLOS ONE style templates and formatting guidelines and revised the manuscript accordingly, including proper formatting of headings and sections, correct file naming conventions, and appropriate structure as per the journal's templates. | Throughout manuscript |
| **Ethics Statement** | 2. In the ethics statement in the Methods, you have specified that verbal consent was obtained. Please provide additional details regarding how this consent was documented and witnessed, and state whether this was approved by the IRB. | Thank you for this important concern. We have revised the Ethics Statement to provide greater detail.  Verbal informed consent was obtained from KII participants. The IRB approval did not specifically prescribe written or verbal informed consent. However, considering the geographically dispersed study settings and the virtual mode of most KIIs, verbal informed consent was obtained and audio-recorded before each interview as a formal documentation. | Page 11, Lines 338-341 |
| **Ethics Statement Location** | 3. Your ethics statement should only appear in the Methods section of your manuscript. | Thank you for reminding us. We confirm that the ethics statement is reported exclusively in the Methods section and does not appear elsewhere in the manuscript. | Page 11, Lines 331-347 |
| **Supporting Information** | 4. Please include captions for your Supporting Information files at the end of your manuscript, and update any in-text citations to match accordingly. | Thank you for pointing out this. Captions for all Supporting Information files have been added at the end of the manuscript, and corresponding in-text citations have been updated. | Page 37-38, Lines 1181-1195 |
| **Citation Recommendations** | 5. If the reviewer comments include a recommendation to cite specific previously published works, please review and evaluate these publications. | We carefully reviewed all reviewer comments and confirmed that no reviewer recommended citation of any specific previously published works beyond reference correction requests. | N/A |

**Response to Reviewer 1**

| **Sub-topic** | **Comments / Suggestions Raised** | **Response / Justification** | **Page & Line Numbers** |
| --- | --- | --- | --- |
| **Overall** | This manuscript addresses an important public health issue and provides useful qualitative evidence on barriers and enablers to HPV vaccination implementation in Ethiopia. The inclusion of multiple stakeholder groups and the use of CFIR 2.0 are strengths. However, several methodological and reporting issues need to be addressed before publication. | We sincerely thank the reviewer for the positive and constructive assessment. We have carefully considered all methodological and reporting issues raised and revised the manuscript accordingly. | Throughout manuscript |
| **Adolescent Participant Age** | 1. The adolescent participants do not align well with the current HPV vaccination target group. The manuscript focuses on girls aged 9-14 years, but the adolescent FGD participants were aged 17-18 years. The authors should justify this sampling decision and acknowledge how it may limit the relevance of adolescent perspectives to the target population. | We thank the reviewer for raising this important methodological issue. We have clarified the rationale for this sampling decision.  To capture first-hand experiences of HPV vaccination, girls aged 17–18 years were purposively selected based on their age and grade. These adolescents were expected to have prior experience of HPV vaccination when they were eligible at age 14 under Ethiopia’s initial single-age cohort, and to express their perspectives on vaccination barriers and facilitators more clearly than younger adolescents.  However, we acknowledge that their perspectives may not fully represent those of the current 9–14-year-old cohorts and this limitation has been explicitly acknowledged in the revised manuscript. | Page 6, Lines 175-180  Page 30, Lines 886-890 |
| **Sampling Bias** | 2. The sampling of adolescent girls may have introduced selection bias. Participants were selected partly based on good academic performance and involvement in school activities, which may have led to the inclusion of more informed or engaged students. The authors should discuss how this may have influenced the findings. | We agree that selecting participants based on academic performance and school activities may have overrepresented more engaged or articulate students and narrowed the range of perspectives. However, purposive sampling aimed to identify information-rich participants rather than achieve statistical representativeness.  We have clarified this rationale in the Methods and acknowledged the potential limitation in the Limitations section. | Page 6, Lines 180-184    Page 30, Lines 882-886 |
| **Contextual Variations** | 3. The study emphasizes diverse socio-ecological contexts, including pastoralist, rural, conflict-affected, urban, and semi-urban settings. However, the results are mostly presented as overall themes. The authors should better show how barriers and enablers varied across regions or context types, or explain why such comparison was not possible. | We thank the reviewer for this crucial observation and fully agree that contextual stratification is important. Our primary objective was to identify cross-cutting barriers and enablers rather than conduct formal regional comparisons.  However, given the diversity of stakeholders represented in the sample, both convergent and divergent perspectives were evident across stakeholder groups. To make these variations more explicit, we have added a new subsection, “Convergent and Divergent Perspectives Across Stakeholder Groups,” to the Results section, highlighting similarities and differences that are directly supported by the data. | Page 14, Lines 372-396 |
| **CFIR Application** | 4. The use of CFIR 2.0 remains relatively superficial. CFIR appears to have been used mainly as an organizing framework, but the manuscript does not clearly show how specific CFIR domains or constructs informed the identification and interpretation of implementation determinants. A mapping table linking themes, subthemes, CFIR constructs, and illustrative quotes would strengthen the analysis. | We thank the reviewer for this important observation.  We have clarified that CFIR 2.0 domains and constructs informed both the development of the data collection tools and the analytic process, particularly the development and refinement of codes and subcategories and the interpretation of implementation determinants. The main categories, however, were developed inductively from patterns emerging in participants’ accounts rather than using CFIR domain names as category labels, allowing the findings to better reflect participants’ perspectives. We have also added **S1 File**, which maps participant quotes, condensed meaning units, codes, subcategories, and main categories to the relevant CFIR 2.0 domains and constructs. | Page 7, Lines 202-204  Page 9-10, Lines 281-303  **S1 File** |
| **Determinant Assessment** | 5. The manuscript does not assess the relative importance, direction, or strength of implementation determinants. If determinant rating was not conducted, this should be stated and acknowledged as a limitation. | We thank the reviewer for this important comment and acknowledge that the analysis did not formally rate or quantify the relative importance of identified barriers and enablers. Rather, it explored stakeholders’ perceptions of these factors and used CFIR 2.0 to understand how and why they influenced implementation within specific contexts. This limitation has been acknowledged in the revised manuscript. | Page 30, Lines 894-896 |
| **Results vs Discussion** | 6. The Results section sometimes contains interpretation that may be better placed in the Discussion. The authors should more clearly separate participants' views from the authors' interpretation. | We thank the reviewer for this helpful observation.  We have revised the Results to more clearly distinguish participants' views and empirically derived findings from the authors' interpretation. We have reduced broader interpretations and explanations in the Results and moved them, where appropriate, to the Discussion. | Throughout Results and Discussion sections |
| **Ethical Procedures** | 7. Ethical procedures for adolescent participants need clearer reporting, particularly guardian consent and adolescent assent for participants under 18 years old, and the approval of audio-recorded verbal consent. | We acknowledge this important concern and revised the Methods/Ethical Considerations section to explicitly describe the consent and assent procedures.  For participants younger than 18 years, informed consent was obtained from parents/guardians, followed by assent from adolescents. For participants aged 18 years, consent was obtained directly. We have granted ethical clearance but it did not specify use of either written consent or audio-recorded verbal consent. Considering the geographically dispersed study settings and the virtual mode of most KIIs, verbal informed consent was obtained and audio-recorded before each interview. | Page 11, Lines 338-345 |
| **Limitations** | 8. The limitations section should more clearly acknowledge the absence of younger girls aged 9-14 years and out-of-school girls, both of whom are highly relevant to HPV vaccination implementation. | We thank the reviewer for raising this important issue.  To capture first-hand experiences of HPV vaccination, girls aged 17–18 years were purposively selected based on their age and grade. These adolescents were expected to have prior experience of HPV vaccination when they were eligible at age 14 under Ethiopia’s initial single-age cohort strategy, and express their perspectives more clearly than younger adolescents.  However, this limitation has been explicitly acknowledged in the revised manuscript. | Page 6, Line 175-180;  Page 30, Lines 885-889 |

**Response to Reviewer 2**

| **Sub-topic** | **Comments / Suggestions Raised** | **Response / Justification** | **Page & Line Numbers** |
| --- | --- | --- | --- |
| **Overall** | Overall, this study is worthwhile and is an important topic, but with the following minor comments. | We sincerely thank the reviewer for the positive and encouraging assessment. We have carefully considered all comments and revised the manuscript accordingly. | Throughout manuscript |
| **Urban Non-Uptake Paradox** | 1. The discussion does not explicitly address the connection between the paradox of left unvaccinated with the parent study's finding. | We agree this connection was not sufficiently explicit. We have revised the final paragraph of the Discussion to make this connection explicit.  The present study provides qualitative insights explaining why urban residence was associated with higher non-uptake in our parent study. Despite better infrastructure and physical access, urban girls—particularly in private and international schools—face context-specific barriers including anti-vaccine misinformation through social media, parental unavailability due to work, delayed consent, and school reluctance to participate in public vaccination programs. | Page 29, Lines 853-863 |
| **Campaign Figures** | 2. Figures should be reconciled (over 6.8 million girls) and that (over 7 million girls) which describe the same campaign in 2024. | We have reviewed the sources and reconciled the figures. The figure of **over 6.8 million girls** has been retained as the more precise estimate, while the rounded figure "over 7 million" has been removed to ensure consistency and avoid ambiguity. | Page 3, Line 82; Page 27, Line 798 |
| **Age Range** | 3. Not the limitation, where there is a controversial statement with girls aged 19-14 but adolescents' studies are aged 17-18 years. | We thank the reviewer for raising this important issue. We have clarified the rationale for this sampling decision.  To capture first-hand experiences of HPV vaccination, girls aged 17–18 years were purposively selected based on their age and grade. These adolescents were expected to have prior experience of HPV vaccination when they were eligible at age 14 under Ethiopia’s initial single-age vaccination strategy, and to express their perspectives on vaccination barriers and facilitators more clearly than younger adolescents.  However, we acknowledge that their perspectives may not fully represent those of the current 9–14-year-old target population and out-of-school girls, and this limitation has been explicitly acknowledged in the revised manuscript. | Page 6, Lines 175-180  Page 30, Lines 885-889 |
| **Rigor Details** | 4. Some claims were asserted but were not substantiated in the results sections and lack supporting details, such as the prolonged engagement, member checking, dense descriptive. | We have revised the manuscript to clarify how rigor was ensured. Specifically, we have provided details on the researchers' engagement with the study setting and participants, the process used for member checking where applicable, and the use of detailed contextual descriptions and verbatim quotations to support the findings. | Page 10-11, Lines 309-330 |
| **References** | 5. Citations should be revised and revisited, reference 6 is dated 2016 but addresses the campaign in 2024, reference 22 and reference 36 are identical, reference 30 should be re-written not as initials, references 29, 38, and 39 are incomplete. | We have avoided one from the duplicate references 22 and 36. Reference 6 has been replaced with an appropriate source for the 2024 campaign. Reference 30 has been corrected. References 29, 38, and 39 have been completed with full bibliographic information. | Reference list, pages 33-38  Line numbers 967-1173 |
| **Participant Codes** | 6. Revisit table 2 where two participants share the same code FGD6P6 while quotations are attributed with different coding. Similarly, table 1. | Thank you for this observation.  We have corrected the error in Table 2, where two participants were assigned “FGD6P6”. The second participant has been corrected to **“FGD6P7.”** | Table 1, page 10;  Table 2, page 13 |
| **Language** | 7. Some English language mistakes were recovered while reading the manuscript, (confusion of was/were) and religousreaders word. Also, there was inconsistent capitalisation of category names. | We have thoroughly proofread the entire manuscript and corrected grammatical errors (including subject-verb agreement), spelling errors (including "religious leaders"), and typographical errors. We have also standardized the capitalization of all qualitative categories and subcategories throughout the manuscript. | Throughout manuscript |

**Response to Reviewer 3**

| **Sub-topic** | **Comments / Suggestions Raised** | **Response / Justification** | **Page & Line Numbers** |
| --- | --- | --- | --- |
| **Overall** | Thank you for the opportunity to review this manuscript. The study addresses an important problem and has clear strengths: an appropriate design (interpretive description) anchored in CFIR 2.0; purposive sampling across four deliberately contrasting settings; an unusually wide stakeholder range, from adolescent girls and religious leaders to ministry-level advisors; instruments that were forward- and back-translated; and a purposeful link to the parent quantitative finding of higher urban non-uptake. The findings are plausible and the recommendations are actionable. | We sincerely appreciate the reviewer’s thoughtful, detailed, and constructive comments and the clear recommendations for strengthening the manuscript. We have carefully considered each suggestion and revised the manuscript accordingly, which has substantially improved its clarity and rigor. | Throughout manuscript |
| **Reporting Guideline** | 1. Reporting guideline missing. No recognized qualitative reporting guideline is used. PLOS ONE expects one under its seventh publication criterion.  Action: Complete the 32-item COREQ checklist (Tong et al., 2007), or SRQR, giving the page and line for each item, and upload it as Supporting Information. Mark any item as not applicable rather than leaving it blank. Add one sentence to the Methods stating that the study is reported in accordance with COREQ. Completing it will also resolve items 5, 10, and Minor 7–8 below. | Thank you for this important suggestion.  We have completed the 32-item COREQ checklist and uploaded it as Supporting Information. We have added one sentence to the Methods (study design subsection) stating that the study is reported in accordance with COREQ. The checklist includes page and line numbers for each item. | Page 5, Lines 149-152;  Completed COREQ checklist uploaded as Supporting Information |
| **Consent Reporting** | 2. Consent reporting incomplete and inconsistent. Verbal consent was used (lines 230–233), but the manuscript does not confirm that the IRB approved an oral procedure or say why written consent was not obtained. The statement that “verbal assent and consent were obtained from participants and their guardians, respectively” leaves “where applicable” undefined and gives no age threshold — relevant because all adolescent participants were 17–18 (Table 2) and the age of majority in Ethiopia is 18. The wording also differs from the submission metadata Ethics Statement.  Action: In the Methods “Ethical approval” section:  – State that the IRB specifically approved verbal consent, and give the reason written consent was waived.  – State which participants were treated as minors and on what basis, and describe separately the guardian-consent-plus-assent process for them and the self-consent process for participants aged 18 and over.  – Describe how individual consent was obtained and recorded in the group setting — whether each participant was recorded agreeing individually, and whether there was a private opportunity to decline.  – Make this section correspond word-for-word with the submission metadata Ethics Statement. | Thank you for these detailed comments and suggestions.  **1. Form of consent and IRB approval:** The study received ethical clearance from the institutional review board (IRB) of the University of Gondar, College of Medicine and Health Sciences, Institute of Public Health (Reference: CMHSSH-UOG IRERC/43/05/2024). The IRB approval did not specifically prescribe written or verbal consent. Given that most KIIs were conducted virtually and the study covered geographically dispersed settings, verbal informed consent was used and audio-recorded before each interview. We have clarified this procedure and the rationale for not obtaining written consent in the revised manuscript.  **2. Consent for adolescent participants:** We have clarified the consent procedures according to participants’ age. Participants aged 17 years were considered minors and participated following guardian consent and adolescent assent, whereas participants aged 18 years provided informed consent themselves.  **3. Consent in focus group discussions:** We have clarified that written informed consent/assent was obtained individually from each FGD participant before the discussion commenced. Each participant was given an opportunity to ask questions and to decline participation.  **4. Consistency with the Ethics Statement:** We have revised the Ethics Statement in the manuscript and the submission metadata to ensure that the wording describing informed consent is consistent across both locations. | Page 11, Lines 331-345  Page 11, Lines 343-345  Page 11, Lines 341-343  Page 11, Lines 331-347 |
| **Data Availability** | 3. Data availability statement non-compliant. The statement at line 776 does not meet the PLOS Data policy. For an interview study the underlying data are the de-identified transcripts or a comprehensive coded-excerpt set; the quotations printed in the manuscript are a selected fraction. Restriction may well be justified here, given small sub-samples and identifiable roles (“traditional healer,” “Edir chairman,” “medical director”) — but it must be stated, not implied.  Action: Replace the statement with either a repository deposit of de-identified transcripts with a DOI, or a restriction statement naming the ethical reason, the approving body, and a non-author contact for access requests. Confirm the wording is consistent with the consent given to participants.  A usable model for the restriction option:  “Data cannot be shared publicly because the transcripts contain potentially identifying information; given the small number of participants and the identifiable roles several informants hold, public release would risk re-identification. De-identified data are available on reasonable request to qualified researchers, subject to approval by the University of Gondar Institute of Public Health IRB (contact: [institutional email]).” | Thank you for this important observation.  We have replaced the Data Availability statement with a restriction statement as follows naming the ethical reason, the approving body, and a contact for access requests.  Data cannot be shared publicly because the transcripts contain potentially identifying information; given the small sub-samples and the identifiable roles several informants hold (“traditional healer,” “Edir chairman,” “medical director”), public release would risk re-identification. De-identified data are available on reasonable request to qualified researchers, subject to approval by the University of Gondar, College of Medicine and Health Sciences, Institute of Public Health IRB (contact: [cmhsshirb2022@gmail.com](mailto:cmhsshirb2022@gmail.com)). | Page 32, Lines 953-959 |
| **Companion Publications** | 4. Companion publications not disclosed. References 12 and 17 appear to be quantitative outputs of the same doctoral project, one of them published in this journal. PLOS ONE’s second criterion and its related-manuscripts policy require this to be visible.  Action: Add a statement to the Methods, and to your cover letter, describing how this qualitative study relates to those papers and confirming no overlap of data or findings. Cite them as companion work at lines 108–112, where the parent study’s urban non-uptake finding is used to motivate this one. | Thank you for highlighting this important issue.  We have added a statement to the Methods section and the cover letter identifying References 12 and 17 (References 19 and 24 in the revised version) as companion publications arising from the same broader doctoral research project. We have also cited these publications where the parent quantitative finding is used to motivate the present qualitative study. We clarify that, although the studies are part of the same doctoral project, they address different research questions and use distinct study components, analyses, and findings, with no overlap in qualitative data, quotations, or reported findings. | Pages 5-6, Lines 154-164;  It is also described in the Cover letter |
| **Reflexivity** | 5. No reflexivity or positionality statement. The principal investigator conducted the interviews and led the analysis, but the manuscript does not report the team’s disciplinary backgrounds, any prior relationship to sites or participants, or their assumptions about HPV vaccination. “Explicit bracketing” is claimed at line 221 but not demonstrated; bracketing is a practice, not a label.  Action: Add a short reflexivity paragraph to the Methods covering: the interviewer’s professional background and qualitative training; how the interviewer was introduced to participants and any pre-existing relationship; the assumptions held about HPV vaccination going in; and the reflexive practices used (for example journaling, memoing, or team debriefing). Use this paragraph to substantiate the bracketing claim, or remove that claim. | Thank you for this important and insightful comment.  We have added a reflexivity and positionality statement to the Methods section describing the interviewer’s professional background and qualitative research training, relationship with participants and study sites, and initial assumptions regarding HPV vaccination. We have also clarified the reflexive practices used during data collection and analysis to minimize the influence of these assumptions on the interpretation of the findings. | Page 9, Lines 260-275 |
| **Participant Region** | 6. Participant region not reported. Four sites were chosen for contrast (lines 117–134) and the access-and-equity findings depend on those contrasts, but Tables 2 and 3 do not record which region each participant came from. Readers cannot confirm all four settings are represented, or judge whether a pastoralist-specific finding rests on one informant or several.  Action: Add a region column to Tables 2 and 3 — region rather than facility, to preserve anonymity — and state the distribution across the four settings in the Results preamble. If any setting is thinly represented, note this as a limit on the claims that depend on it. | Thank you for this valuable suggestion.  We agree that reporting participants’ region provides important contextual information and helps demonstrate the geographical diversity of the qualitative sample. Accordingly, we have added a “Region/city administration” column to the participant characteristics in table 1 and 2 in the revised manuscript. | Tables 2 and 3, pages 12-13 |
| **Sampling Limitations** | 7. Sampling limitations not acknowledged. Adolescent participants were recruited through schools and selected partly for good academic performance and extracurricular involvement (lines 148–151); all were 17–18 (Table 2), older than the 9–14 cohort now vaccinated; and out-of-school, pastoralist and private-school girls — the groups the study identifies as underserved — are absent from the focus groups, reaching the reader only through providers and administrators. I recognise that interviewing 9–14-year-olds carries its own ethical burden and may have been deliberately avoided; the remedy is transparency, not re-sampling.  Action: Add these three points to Strengths and Limitations, state the likely direction of the effect (barriers among the hardest-to-reach are probably understated), and note what they imply for interpreting the urban non-uptake paradox. Where a finding rests on groups not directly sampled, attribute it in the Results to provider or administrator accounts rather than to girls or families. | Thank you for this important and constructive comment. We thank also for understanding the ethical burden of interviewing 9–14-year girls.  We have extended the Limitations section to explicitly acknowledge that adolescent participants were recruited through schools and were selected partly based on academic performance and extracurricular involvement, that all adolescent participants were aged 17–18 years, and that out-of-school, pastoralist, and private-school girls were not directly represented in the FGDs. We now acknowledge that barriers affecting the hardest-to-reach groups may therefore be underestimated and that this limitation should be considered when interpreting the findings. | Page 30, Lines 881-891 |
| **Paradigm vs Reliability** | 8. Paradigm and reliability measure conflict. The stated “constructivist and relativist ontologies” (lines 140–143) sit awkwardly with directed content analysis using a priori categories and with an inter-coder reliability statistic, since reliability-as-convergence presupposes a correct coding. Separately, 84% raw percent agreement (lines 202–205) ignores chance agreement and cannot support the claim of “strong” reliability; nor is percent agreement a statistical test, as line 202 states.  Action: Choose one of two routes and apply it consistently:  – Option A — reposition the paradigm as pragmatic or naturalistic (which interpretive description accommodates), and report a chance-corrected coefficient (Cohen’s kappa, or Brennan–Prediger or Gwet’s AC1, which are more robust when code prevalence is skewed) with its value and interpretation.  – Option B — keep the relativist framing, remove the statistic, and describe the two-coder exercise as a consensus or negotiated-agreement process, stating how disagreements were resolved.  Action: Also correct the wording at line 202, which describes percent agreement as having been “statistically tested.” | Thank you for this important methodological observation.  We keep the relativist framing and removed the 84% raw agreement statistic. Instead, we describe the two-coder exercise as a consensus-oriented process used to examine and refine the coding framework. Differences in coding were discussed between the researchers and resolved through discussion and agreement, while the final interpretation remained grounded in participants’ accounts. | Page 10, Lines 297-301 |
| **CFIR Mapping** | 9. CFIR mapping absent and citation incorrect. The analysis is described as guided by CFIR 2.0, but the Results categories are never linked to CFIR’s five domains and constructs, so the framework claim cannot be verified. Reference 20 is the CFIR Outcomes Addendum rather than the updated framework, and its own details are wrong (given as 2022;5:1–10).  Action: Add a table mapping each category and subcategory to its CFIR 2.0 domain and construct, noting any domains not represented and why. Replace the framework citation with: Damschroder LJ, Reardon CM, Widerquist MAO, Lowery J. The updated Consolidated Framework for Implementation Research based on user feedback. Implementation Science. 2022; 17:75. Retain the Addendum (correctly cited as Implementation Science 2022;17:7) only if outcomes conceptualisation is actually used. | We appreciate the reviewers for highlighting these important points. In response, we have added Supporting file 1 (S1_File), which maps each code and subcategory to its corresponding CFIR 2.0 domain and construct. It is important to note, however, that not all CFIR domains and constructs are represented in our analysis, as some were not directly relevant to the emerging themes identified in our data. We intentionally chose to use our own category names rather than adopting the CFIR domain labels directly, to ensure that our categories remained grounded in and reflective of the participants' own expressions.    We have replaced the framework citation with the correct Damschroder et al. 2022 Implementation Science reference. | Supporting_ file 1 (S1_file);  Reference 28 in the revised version, page 34 |
| **Interview Language** | 10. Interview and coding language unclear. The instruments were properly forward- and back-translated (lines 173–175) and transcripts were produced in Amharic then translated (line 195), but the language of the interviews themselves is not stated. Afar and parts of Dire Dawa have substantial non-Amharic-speaking populations, so this bears directly on the equity findings.  Action: State in the Methods which language(s) the interviews were conducted in and, if Amharic only, how this was managed and whether it affected participation in pastoralist settings. State whether coding was done on the Amharic or English transcripts, and what preserved meaning through translation for the quotations presented. | Thank you for raising this concern.  We have stated in the Methods that interviews were conducted in Amharic as it is the official language in Amhara, Addis Ababa and Dire Dawa and all participants were able to speak it (we purposively selected bilingual participants in Afar). Coding was done on the English translated transcripts. We also explain the quality-control procedures used to preserve meaning during translation, including back-translation of the interview guides, verification of transcripts against audio recordings, and review of ambiguous passages. | Methods; Data Collection Procedures;  Page 8; Lines 228-244 |
| **Interpretive Claims** | 11. Interpretive claims exceed the design. Passages such as confidence in evidence “plays a critical role in sustaining implementation efforts beyond initial rollout” and perceptions that “reduced hesitancy among frontline providers” (lines 279–282) assert causal and temporal effects that one-off interviews cannot establish.  Action: Rephrase these as interpretations consistent with participant accounts rather than demonstrated effects, and move forward-looking sustainability inferences to the Discussion. Apply the same edit wherever the Results assert that a factor caused, reduced or sustained an outcome. | Thank you for this important observation.  We revised the Results to explicitly attribute interpretations to participants’ accounts and avoid causal or temporal claims. Statements on provider hesitancy, sustained implementation, and programme sustainability were rephrased or moved to the Discussion as implications. Similar statements were systematically revised using terms such as “participants described,” “participants perceived,” and “participants reported.” | Throughout Results and Discussion sections |
| **Reported Figures** | 12. Reported figures and supporting citations need correction.  Four checkable problems:  – Line 248 states that half (50%) of discussants were Orthodox Christian. Recomputing Table 2 gives 20 of 42 Orthodox (47.6%), 21 Muslim (50.0%) and 1 Protestant (2.4%) – the two figures appear transposed, noting that 47.6% is correctly used in the same sentence for education. All other reported descriptives verify correctly.  – Reference 6 (2016) is cited at line 74 for the 2024 single-dose transition and at line 654 for the multi-age-cohort campaign reach. A 2016 source cannot document 2024 events.  – Lines 94–97 give approximately 970,000 cases averted and nearly 932,000 lives saved, which together imply a case-fatality ratio near 96%.  – Campaign reach appears as “over 6.8 million” (line 75) and “over 7 million” (line 654); the “at least 13 million” cumulative figure (line 76) is unsourced.  Action: Correct the religion percentages against Table 2; cite contemporaneous sources for the 2024 policy change and campaign reach; verify both modelling figures against references 15 and 16 and state precisely what each represents, including scenario and time horizon; and use one consistently rounded reach figure with its source throughout. | Thank you for identifying this error.  We rechecked Table 2 and corrected the reported religious distribution.  We have cited contemporaneous sources for the 2024 policy change and campaign reach.  We have verified both modelling figures against references 15 and 16 (22 and 23 in the revised version).  We use one consistently rounded reach figure throughout. Reference is also added to the unsourced text. | Page 12, Line 356  Reference 12 in the revised version; Page 33, Lines 998- 1002  Page 4; Lines 104-105  Page 3; Lines 81-83 and page 27; Line 798 |
| **Minor Comments** | | | |
| **Quotation Fidelity** | 1. Quotation fidelity. The quotation preferring the vaccine to screening is attributed to FGD3P5 at line 293 but to FGD2P5 in Table 1, with different wording in each; per Table 2 these are very different participants. The KII-1 effectiveness quotation is also worded differently in Table 1 and at lines 283–284.  Action: Check both against the transcripts, correct the attribution and wording, reproduce each quotation identically wherever it appears, and audit the remaining quotations against Table 2. | Thank you for pointing out this.  We rechecked the relevant quotations against the original audio recordings/transcripts and corrected discrepancies in wording and participant attribution. We also conducted a broader audit of quotations throughout the manuscript and tables 1 and 2 to ensure that each quotation is reproduced consistently and attributed to the correct participant code. | Table 1 (page 10)  Table 2  pages 12-13; Lines 420-421 |
| **Participant Code** | 2. Duplicated participant code. Table 2 rows 41 and 42 are both coded FGD6P6; the second is presumably FGD6P7.  Action: Correct the code and confirm both the FGD6 count and the total of 42. | Thank you for pointing this duplicate code. The second FGD6P6 has been corrected to FGD6P7. We confirmed that FGD6 contains seven participants and that the total number of FGD participants remains 42. | Table 2, page 12-13 |
| **Category Labels** | 3. Category labels vary between sections. For example, “Health system resource constraints” (Abstract) against “Health System Resource Constraints and Coordination Gaps” (Results), and “Access constraints and equity gaps” against “Structural Access Constraints and Equity Gaps.”  Action: Use identical category names in the Abstract, Results and Discussion, since the category set is the study’s main contribution. | We are deeply grateful to the reviewer for catching this inconsistency. We completely agree that uniform terminology is essential.  To address this, we have carefully gone through the Abstract, Results, and Discussion sections and have now standardized all category labels to ensure they are perfectly identical across the entire manuscript. | **Abstract** (page 2, Lines 45-51  **Results** (page 15-18, Line 403-514  **Discussion**  (Page 26, Line numbers 747-753 |
| **Barrier Category** | 4. Barrier Category 1 departs from the reporting pattern. Every other category declares its subcategories; this one does not, although it contains two distinct strands — target-group suspicion and consent administration — which also makes its heading unwieldy.  Action: Either declare its subcategories in the same style as the other categories, or split it into two categories with shorter headings. | Thank you for showing us the departure.  We have declared its subcategories in the same style as the other categories. | Pages 20 Lines 565-586 |
| **FGD Composition** | 5. Focus-group composition unexplained. FGD3–FGD4 mixed genders and FGD5 combined Orthodox, Muslim and Protestant leaders, on a topic involving adolescent sexuality and fertility.  Action: State the rationale for these compositions in the Methods and note any effect on candour in the limitations, alongside the social-desirability point already made. | Thank you for this important observation.  We have clarified the rationale for the FGD composition in the Method section. Mixed-gender FGD3–FGD4 captured diverse perspectives from parents and community members, while FGD5 included religious leaders from different faith traditions.  We also acknowledge in the Limitations that these compositions may have affected openness in discussing sensitive issues and introduced social-desirability effects. | Page 6, Lines 169-173  Page 30, Lines 892-894 |
| **"Parents" Definition** | 6. “Parents” not established as a criterion. The Abstract calls FGD3–FGD4 participants parents; the Results describe married adults including teachers, merchants, housewives and a guard.  Action: Confirm whether parental status — ideally of an age-eligible daughter — was an inclusion criterion, and use consistent terminology throughout. | Thank you for this important observation. We have clarified that being parents of an age-eligible daughter was an inclusion criterion for FGD3–FGD4. These participants were adults who had girls eligible for HPV vaccination and were purposively selected to provide perspectives on HPV vaccination and its implementation. We have revised in the abstract, methods and results accordingly and used the term “parents” consistently. | Abstract  (Page 2, Lines 36-37)  Methods (Page 6, Line 168)  Result  (Page 12, Line 353) |
| **Saturation** | 7. Saturation not described. Sample size is attributed to information saturation (lines 164–167) without describing the process.  Action: State in one or two sentences how saturation was judged and documented, separately for focus groups and key-informant interviews, including whether analysis ran in parallel with data collection. | Thank you for raising this point. We clarified that data collection and preliminary analysis were conducted iteratively. Saturation was considered reached when consecutive FGDs and KIIs generated no substantially new codes, concepts, or perspectives. This was documented through ongoing coding, analytic notes, and team discussions, and the Methods section has been revised accordingly. | Page 8, Lines 246-255 |
| **Trustworthiness** | 8. Trustworthiness claims unsupported. “Extensive prolonged engagement… prior to data collection” (line 214) and member checking (lines 216–217) are asserted but not described, with fieldwork spanning four regions in roughly ten weeks.  Action: Specify what prolonged engagement involved and how long it lasted, and state with whom member checking was done, on what material, and how the feedback changed the analysis. | Thank you for this essential comment. We have clarified that prolonged engagement included the PI’s one-month internship attachment at the Ministry of Health before data collection, which facilitated familiarity with the study context and interactions with relevant KII participants, complemented by repeated engagement during data collection. We also specified that member checking involved sharing preliminary conceptual summaries with selected key informants and incorporating their feedback to clarify meanings and refine emerging interpretations. These details have been added to the Methods section. | Pages 10-11, Lines 309-330 |
| **Analytic Approach** | 9. Analytic approach imprecisely named. The “hybrid coding structure” combining deductive CFIR categories with inductive codes (lines 199–201) is a combined deductive–inductive analysis rather than directed content analysis in the strict Hsieh and Shannon sense.  Action: Name the approach precisely and use the same term consistently throughout the Methods, Results and Discussion. | Thank you for this important observation. We have revised the manuscript to consistently describe the approach as combined deductive–inductive content analysis guided by CFIR 2.0. | Abstract  (Page 2, Lines 40-41)  Methods (Page 9, Lines 281-282) |
| **Limitations** | 10. Limitations section too narrow. It currently acknowledges only social-desirability bias.  Action: Expand it to cover the school-based and performance-weighted adolescent sampling; the absence of out-of-school, pastoralist, private-school and 9–14-year-old participants; the age gap between participants and the current target cohort; any uneven coverage across the four settings; and translation effects. State the likely direction of each.  **Editorial and Language**  – Replace the recurring construction “was/were emerged” with “emerged” (for example lines 427, 549, 659).  – Correct: “may still influenced responses” (line 725) to “may still have influenced”; “schedules lately” (line 552) to “late”; “religiousleaders” (line 673) to “religious leaders.” A full professional proofread is advised.  – Regenerate the reference list from your reference manager and check every entry. References 22 and 36 are the same Hall et al. paper cited twice — removing the duplicate will renumber the list. References 28 and 42 begin with ORCID artefacts (“Id KH…”, “Id DG, Id DW…”); reference 23 embeds JSTOR metadata; reference 26 carries “HHS Public Access” as its title; reference 30 parses title words as author initials (“Strategy G, Accelerate TO, Elimination THE…”); reference 50 embeds “iMedPub Journals” and “Abstract”; reference 2 inverts the author name (“Martel C De”) and omits the journal; references 8, 29, 38 and 47 lack journal names, years or other essential details. Verify all names, titles, journals, years and DOIs against PLOS ONE style, and confirm every in-text citation resolves to the intended source.  – Attach a citation to the figure of 140 countries having introduced HPV vaccination as of 2024 (lines 62–63) and confirm it against the source year.  – Confirm all abbreviations are defined at first use in both the abstract and the main text, and that the abstract is within the journal’s word limit. | Thank you for these detailed editorial and language suggestions.  We have expanded the limitations it to cover: school-based and performance-based adolescent sampling; absence of out-of-school, pastoralist, private-school and 9-14-year-old participants; age gap between participants and current target cohort; uneven coverage across settings; and translation effects. We state the likely direction of each.  We have carefully revised the manuscript accordingly. Specifically, we corrected the identified grammatical and spacing errors and conducted a thorough language review.  We also regenerated and checked the reference list, removed the duplicate reference, corrected metadata and author-name artefacts, completed missing bibliographic details, verified DOIs and in-text citations, and ensured consistency with PLOS ONE style.  A citation has been added to support the statement regarding the 140 countries (the figure was also edited as 147 countries) introducing HPV vaccination by 2024, and the source year was verified.  We also checked that all abbreviations are defined at first use in both the abstract and main text and confirmed that the abstract complies with the journal’s word limit. | Page 30, Lines 881-891  Throughout the results and discussion sections.  Examples:  Page 408-411, Line 414-419  Page 18, Line 522-529  Throughout the whole references (pages 33-37)  Page 3, Line 64  Throughout the manuscript |

**Response to Reviewer 4**

| **Sub-topic** | **Comments / Suggestions Raised** | **Response / Justification** | **Page & Line Numbers** |
| --- | --- | --- | --- |
| **Title** | The title clearly indicates this is a qualitative study and reflects the study's aim. Consider including the study population under study in the title. | Thank you for this helpful suggestion. We agree that specifying the study population in the title would provide greater clarity regarding the scope of the study. As the study explored perspectives from multiple stakeholder groups involved in HPV vaccination implementation—including adolescent girls, parents, community and religious representatives, healthcare providers, immunization coordinators, administrators, and technical advisors—we have revised the title to explicitly indicate that the study was conducted among key stakeholders: **"Enablers and Barriers to Human Papillomavirus Vaccination Implementation Among key stakeholders in Ethiopia: A Qualitative Study"** | Title page |
| **Abstract** | Reports a clear brief background, study objective is clear. Main findings summarised as themes, conclusions consistent. | We thank the reviewer for this positive assessment. | Page 2, Lines 28-58 |
| **Introduction** | The authors have mentioned the importance of HPV vaccination. The burden of HPV-related disease in the population being studied has not been clearly mentioned. The authors should quantify the problem, provide figures to support this. | Thank you for this important suggestion.  We have strengthened the introduction. Specifically, we now report that burden of cervical cancer increased substantially between 2010 and 2020, with annual cases rising from 4,648 to 7,445 and deaths from 3,235 to 5,338, while the number of women aged ≥15 at risk increased from 20.9 to 36.9 million [7–9]. The estimated annual incidence and mortality rates were 35.9 and 22.6 per 100,000 women, respectively, among the highest in the region. | Page 3, Lines 73-78 |
| **Methods - Study Settings** | Line 129: "In Ethiopia, the HPV vaccine is mainly delivered through school-based programmes..." This statement should probably be in the introduction. | We agree and have moved this sentence to the Introduction section. | Page 3, Lines 88-90 |
| **Methods - Study Design** | A qualitative descriptive study design is appropriate. The authors also provided a clear justification for their choice of study design. | Thank you for this positive assessment. We understand the reviewer want to say interpretive descriptive and appreciate the recognition that the choice of design was appropriate and clearly justified. | Page 5, Lines 141-146 |
| **Methods - Sampling** | Although the authors purposively selected adolescent girls who were considered information-rich participants, it is unclear whether the sample was sufficiently diverse. | Thank you for this valuable comment. We purposively selected diverse stakeholders, including adolescent girls, parents, community and religious leaders, healthcare providers, and administrators from governmental and non-governmental organizations to obtain rich and varied perspectives.  We acknowledge the underrepresentation of students who were less engaged in academic or extracurricular activities, as well as out-of-school and private-school students, as a limitation of the study. | Page 6, Lines 165-173 and 186-190  Page 30, Lines 881-891 |
| **Methods - Data Collection** | - CFIR framework was used; this is a strength. - It may be more systematic to distinguish between data collection tools and procedures under separate subheadings.  - The term "kebele" should be defined. | - Thank you for pointing out this.  -We agree and have separated Data Collection Tools and Data Collection Procedures into distinct subheadings.  - We have defined "kebele" as the smallest administrative unit in Ethiopia. | Page 7, Line 200 and 210  Page 7, Line 219 |
| **Results** | Theme development was appropriate. Researchers interpreted underlying patterns and explained relevance. Researchers however did not clearly identify where perspectives in KII or FGD agreed or differed. | We sincerely thank the reviewer for this constructive and insightful observation. We have added paragraphs in the result section after tables 2 and 3 describing areas of agreement and divergence between participant groups (FGD vs KII perspectives). | Page 14, Lines 372-396 |
| **Discussion** | The findings answer the research question and the authors managed to derive meaning behind their findings and considered cultural and social contextual factors. | Thank you for this positive and encouraging assessment. We appreciate the reviewer’s recognition. |  |
| **Strengths and Limitations** | The authors did not comment or report if reflexivity could have been a methodological weakness. Potential sampling limitation noted. | Thank you for this important and insightful comment.  In the revised version, we have added a reflexivity and positionality statement to the Methods section describing the interviewer’s professional background and qualitative research training, relationship with participants and study sites, and initial assumptions regarding HPV vaccination.  We have also added the potential sampling limitations and their implications for the transferability of the findings in the revised manuscript. | Page 9, Lines 260-275  Page 30, Lines 898-899 |

**Response to Reviewer 5**

| **Sub-topic** | **Comments / Suggestions Raised** | **Response / Justification** | **Page & Line Numbers** |
| --- | --- | --- | --- |
| **Overall** | This is a timely and policy-relevant qualitative study addressing barriers and facilitators to HPV vaccine implementation in Ethiopia. The use of multiple stakeholder groups and the CFIR 2.0 framework are important strengths. Nevertheless, several methodological and reporting issues should be addressed to improve transparency, rigor and reproducibility. | We sincerely thank the reviewer for the constructive assessment. We have carefully considered all comments and strengthened the manuscript accordingly. | Throughout manuscript |
| **Sampling Justification** | 1. Sampling strategy requires better justification. Adolescent girls selected according to academic performance and extracurricular activities may have preferentially included highly engaged students while excluding girls who are less connected to school. | Thank you for your insightful comment. In the methods section we clarified that information-rich participants were purposively selected to obtain detailed insights.  However, we acknowledge that selecting participants based on academic engagement and school activities may have limited the diversity of perspectives. This potential limitation has been acknowledged in the Limitations section. | Page 6, Lines 180-184  Page 30, Lines 881-885 |
| **Saturation Documentation** | 2. Saturation is insufficiently documented. No explanation is provided regarding how saturation was assessed, who evaluated it, whether code saturation or meaning saturation was considered, or whether saturation was reached separately for each stakeholder group. | We have described how saturation was assessed and documented, separately for focus groups and key-informant interviews. Analysis ran in parallel with data collection, and saturation was evaluated by the principal investigator in consultation with the research team. Both code saturation and meaning saturation were considered. | Page 8, Lines 246-255 |
| **Analysis Detail** | 3. Qualitative analysis requires greater methodological detail. The description of directed content analysis remains rather generic. | Thank you for this helpful comment.  Based on one of the reviewer comments, we updated/modified the analysis method to **‘’combined deductive–inductive content analysis guided by CFIR 2.0.’’** We have clarified: how inductive and deductive coding interacted (hybrid approach); how disagreements between coders were resolved (consensus through discussion); whether coding evolved during data collection and analysis (iterative refinement); and whether reflexive discussions occurred during category development (regular team debriefings). | Page 9-10, Lines 276-305 |
| **Results vs Discussion** | 4. Results and Discussion occasionally overlap. Several sections of the Results include substantial interpretation, while the Discussion sometimes repeats descriptive findings. | Thank you for raising this important suggestion. We have revised the Results to more clearly present participants' perspectives with minimal interpretation, and moved broader interpretations to the Discussion. | Throughout Results and Discussion |
| **Novelty** | 5. Novelty should be emphasized. Many identified barriers have already been described in previous studies from low-income countries. | Thank you for this important concern.  Although some of our findings are similar with those reported in previous studies, our study differs both methodologically and substantively. Methodologically, we applied CFIR 2.0 to examine interacting determinants of HPV vaccination implementation across multiple levels and triangulated perspectives from a broad range of key stakeholders, including adolescent girls, parents, community representatives (community and religious leaders), health workers, and administrators from both governmental and non-governmental organizations.  Substantively, the qualitative inquiry was informed by our preceding quantitative finding of higher non-uptake in urban areas, which enabled us to identify urban-specific barriers, particularly those affecting private and international schools, as well as challenges related to pastoralist populations.  Overall, the use of CFIR 2.0, together with the triangulation of perspectives across stakeholder groups and the linkage between quantitative and qualitative findings, enabled us to generate additional implementation insights by examining how multilevel factors interact within the Ethiopian context, thereby extending rather than simply replicating findings from previous studies. | Page 6, Lines 165-173; 186-190  Pages 5-6, Lines 154-164  Result (page 25, lines 719-734) |
| **Minor Comments** | | | |
| **Participant Description** | 1. Demographic tables are comprehensive but quite detailed. Consider moving part to Supplementary Material. | Thank you for this valuable suggestion.  We carefully considered whether the level of detail in the participant characteristics table could be reduced.  However, in a qualitative study a detailed description of the sample enhances transparency, contextual understanding, trustworthiness and transferability of the findings. Specifically, the reported characteristics—including age, sex, religion, education, occupational status, professional experience, profession, organization, and region—provide important information about the diversity and composition of the participants and help readers assess the contextual relevance of the findings. Nevertheless, we carefully reviewed the table for any information that could potentially expose or identify individual participants or compromise their confidentiality. We believe this approach provides an appropriate balance between comprehensive reporting to support qualitative rigor and the protection of participants’ privacy. | Pages 12-13; Tables 2 and 3 |
| **Language** | 2. The manuscript is generally well written. Minor English editing recommended. | We have thoroughly proofread the entire manuscript and corrected grammatical errors, awkward phrasing, and inconsistent capitalization. | Throughout manuscript |
| **Recommendations** | 3. Recommendations section is useful but somewhat prescriptive. Some recommendations extend beyond what qualitative findings alone can directly support. | Thank you for this important observation. We have revised the recommendations to more closely reflect the barriers and facilitators identified by participants and to frame specific interventions as potential options for consideration rather than definitive policy prescriptions. The revised recommendations emphasize evidence-informed priorities for improving HPV vaccination implementation and equity in the Ethiopian context. | Page 31, Lines 913-939 |

We once again thank the Editor and all reviewers for their insightful and constructive feedback, which has significantly improved the clarity, rigor, and overall quality of the manuscript. We believe the revisions have addressed all concerns raised and look forward to your favourable consideration of the revised manuscript.
