# Supplementary material for: Enablers and barriers to human papillomavirus vaccination implementation among key stakeholders in Ethiopia: A qualitative study
Source: PLoS One. 2026 Nov 2;21(11):e0361925. doi: 10.1371/journal.pone.0361925 (PMC13726970; doi:10.1371/journal.pone.0361925)
Supplement: Supplementary file 5 [file pone.0361925.s006.docx]

**Point-by-Point Response to the academic Editor and Reviewers**

**Manuscript Title:** **Enablers and Barriers to Human Papillomavirus Vaccination Implementation among key stakeholders in Ethiopia: A Qualitative Study**

We sincerely thank the Editor and reviewers for their constructive and valuable comments on our manuscript. We also appreciate their acknowledgment that our previous revisions adequately addressed the comments and suggestions raised during the earlier review process. We are grateful for the opportunity to further improve the manuscript through this minor revision. We have carefully considered each of the remaining comments and made the necessary revisions to enhance the clarity and quality of the manuscript. Below, we provide a point-by-point response to the comments raised, indicating the corresponding changes and page and line numbers in the revised manuscript.

**Response to the Academic Editor**

| **Sub-topic** | **Comments / Suggestions Raised** | **Response / Justification** | **Page & Line Numbers** |
| --- | --- | --- | --- |
| **Journal Requirements** | 1. If the reviewer comments include a recommendation to cite specific previously published works, please review and evaluate these publications to determine whether they are relevant and should be cited. There is no requirement to cite these works unless the editor has indicated otherwise. | We thank the Editor and the journal for this clarification. We carefully reviewed the comments provided by the Academic Editor and reviewers and confirmed that none of them recommended citing specific previously published works. Therefore, no additional references were required on this basis. | Not applicable |
|  | 2. Please review your reference list to ensure that it is complete and correct. If you have cited papers that have been retracted, please include the rationale for doing so in the manuscript text, or remove these references and replace them with relevant current references. Any changes to the reference list should be mentioned in the rebuttal letter that accompanies your revised manuscript. If you need to cite a retracted article, indicate the article’s retracted status in the References list and also include a citation and full reference for the retraction notice. | We thank the Editor and the journal for this important reminder. We carefully reviewed the reference list to ensure its completeness and accuracy, and added some missed bibliographic details for some references where necessary. We also verified that the manuscript does not cite any retracted publications. These corrections have been incorporated into the revised manuscript to improve the accuracy and consistency of the reference.  . | Page 33-37, Lines 967-1183 |

**Response to Reviewer 1**

| **Concern** | **Comments / Suggestions Raised** | **Response / Justification** | **Page & Line Numbers** |
| --- | --- | --- | --- |
| **Overall** | The authors have substantially revised the manuscript and adequately addressed most of my previous comments. The manuscript has improved considerably. I have only the following remaining comments before acceptance. | We sincerely thank the reviewer for the positive assessment of our revised manuscript and for acknowledging the improvements made in response to the previous comments. We greatly appreciate the reviewer’s constructive feedback and the opportunity to address the remaining comments. We have carefully considered each of the additional comments and revised the manuscript accordingly. |  |
| **Clarification of IRB Approval for Audio-Recorded Verbal Consent** | 1. The response states that the IRB approval did not specifically prescribe written or verbal consent. This does not fully clarify whether audio-recorded verbal consent for the KIIs was included in the approved protocol or subsequently confirmed by the IRB. Please provide an explicit statement on this point. | Thank you for this clarification. Sorry for the ambiguity we create in our previous response. We confirm explicitly that audio-recorded verbal consent for the KIIs was included in the study protocol submitted to and approved by the CMHSSH IRERC. The IRB approval letter dated July 2024 grants ethical clearance for the study as described in that approved protocol. Therefore, the audio-recorded verbal consent procedure was part of the original IRB-approved protocol and was not a later amendment, deviation, or separate confirmation. We have added this explicit statement to the Methods section of the revised manuscript. | Page 11, Lines 341-342 |
| **Contextual Differences in Barriers and Facilitators and Sampling Limitations in Afar** | 2. The new subsection on contextual differences is useful, but the stakeholder groups and geographical settings were not fully crossed, and participants in Afar were restricted to Amharic-speaking bilingual individuals. The reported rural, pastoralist, and urban differences should therefore be presented as patterns observed in the available accounts rather than as systematic regional comparisons. The potential exclusion of non-Amharic-speaking participants should also be acknowledged. | We thank the reviewer for this insightful observation. We revised the subsection to clarify that rural, pastoralist, and urban differences represent patterns emerging from the available participant accounts rather than systematic comparisons across geographical settings, given that stakeholder groups and locations were not fully cross-represented.  We also acknowledged in the limitation section that restricting participant recruitment in Afar to Amharic-speaking bilingual individuals may not represent non-Amharic-speaking community members, potentially limiting the representation of their sociocultural perspectives and experiences in the findings. | Page 14, Lines 373-395  Page 30, Lines 898-900 |
| **Clarification of Participants’ Prior Eligibility and Exposure to the HPV Vaccination Programme** | 3. The manuscript states that girls aged 17–18 years were selected to capture “first-hand experiences” of HPV vaccination. Please clarify whether their vaccination status or actual participation in the earlier vaccination programme was confirmed. If not, the wording should refer to prior eligibility or potential programme exposure rather than confirmed vaccination experience. | We thank the reviewer for this important clarification. The vaccination status and actual participation of girls aged 17–18 years in the earlier vaccination programme were not independently confirmed for the purpose of establishing prior vaccination experience. We have therefore revised the wording to refer to their prior eligibility or potential exposure to the HPV vaccination programme rather than implying confirmed vaccination experience. | Page 6, Lines 176-182 |

**Response to Reviewer 4**

| **Sub-topic** | **Comments / Suggestions Raised** | **Response / Justification** | **Page & Line Numbers** |
| --- | --- | --- | --- |
| **Overall** | The authors have addressed my comments and those of the other reviewers appropriately and satisfactorily. I am satisfied with the revisions made. | We sincerely thank the reviewer for the positive evaluation of our revised manuscript and for acknowledging that we have appropriately and satisfactorily addressed the comments raised during the review process. We greatly appreciate the reviewer’s time, constructive feedback, and recognition of our efforts to improve the manuscript. | Not applicable |
